# Evaluating a Website on Learning Disorders for Parents and Learning Therapists: Observational Mixed Methods Study

**DOI:** 10.2196/68365

**Published:** 2025-09-26

**Authors:** Olga Hermansson, Paula Dümig, Björn Witzel, Lior Weinreich, Susanne Volkmer, Gerd Schulte-Körne, Kristina Moll

**Affiliations:** 1 Department of Child and Adolescent Psychiatry University Hospital Ludwig-Maximilians-University Munich Munich Germany

**Keywords:** website evaluation, public health education, internet research, RE-AIM framework, information system continuance intention

## Abstract

**Background:**

Between 5% and 15% of children worldwide have a specific learning disorder. This creates a substantial demand for information among both parents and professionals. LONDI (londi.de) is a German-language website that (1) provides evidence-based information on learning disorders and (2) offers a tool to search for relevant diagnostics and intervention measures for professionals (Help System). This paper reports the results of an evaluation study of the website.

**Objective:**

The aim of the study was to (1) evaluate the website and (2) inform existing theories on technology acceptance and user satisfaction. The study was conducted using the RE-AIM framework for evaluating public health impact and the information system continuance intention framework.

**Methods:**

This mixed methods observational study was conducted online from February 2023 to August 2023 in Germany. Parents of children with learning difficulties and learning therapists participated in a 1.5-hour online session in which they were guided through the website. A pre-post design was used to assess changes in participants’ knowledge, attitudes, and self-efficacy. Additionally, two path models assessing the predicting factors of the intention to further use (ie, continuance intention) and the intention to recommend the website were tested. The first model was based on the widely used information system continuance intention framework and tested expectations confirmation, perceived usefulness, and website satisfaction as predictors of the continuance intention and the intention to recommend the website. The second model focused on usability, content perception, visual aesthetics, and satisfaction as predictors of the same outcome variables.

**Results:**

A total of 77 parents and 73 learning therapists participated in the study. In the sample of learning therapists, age correlated negatively with usability opinion and website satisfaction. A 2-tailed *t* test revealed a significant increase in knowledge about learning disorders in both groups (parents: *t*_76_=12.02, *P*<.001; learning therapists: *t*_71_=7.03, *P*<.001). There was no change in attitudes and self-efficacy in parents (*F*_1,76_=2.04, *P*=.14; Wilks lambda=0.95), but there was a significant change for learning therapists (*F*_1,68_=15.83, *P*<.001; Wilks lambda=0.68) after using the website. A path analysis revealed that the intention to recommend the website can be included as an additional variable in the information system continuance intention framework. For the informational pages, content perception and visual aesthetics significantly predicted website satisfaction (*R*^2^=0.59, *F*_3,143_=69.06, *P*<.001), and content perception significantly predicted continuance intention (*R*^2^=0.45, *F*_3,143_=39.74, *P*<.001). For the Help System, usability was the only significant predictor of website satisfaction (*R*^2^=0.45, *F*_2,67_=28.16, *P*<.001), continuance intention (*R*^2^=0.34, *F*_1,68_=34.57, *P*<.001), and intention to recommend (*R*^2^=0.21, *F*_1,68_=19.02, *P*<.001).

**Conclusions:**

The website has been evaluated positively and has proven useful for the target audience. Predictors of website acceptance and further use are contextual and depend on the website type.

## Introduction

### Background

Specific learning disorder is defined as persistent impairment in at least one of the three learning domains: reading, spelling, and mathematics [1]. They are often referred to as dyslexia or dyscalculia, depending on the affected skill area. People affected by dyslexia typically have problems with reading accuracy and fluency and with spelling; people affected by dyscalculia have problems with understanding and processing numerical information, resulting in problems with memorizing arithmetic facts and with calculations [1,2]. Learning disorders are not due to other conditions, such as intellectual disability, neurological conditions, or lack of instruction [3]. Between 5% and 15% of children worldwide have a specific learning disorder, according to the Diagnostic and Statistical Manual of Mental Disorders (Fifth Edition; DSM-5) [1]. However, the prevalence of undiagnosed learning difficulties in at least one of the three learning domains (reading, spelling, and mathematics) is estimated to be much higher (eg, 23% in Germany and approximately 20% in the United States) [4,5]. The consequences of learning disorders are worrisome: Academic failure, psychiatric disorders, and difficulty participating in society are some of the many risks that such children face in later life [6-8].

Consequently, a question arises: Where do caretakers and professionals turn for reliable, evidence-based information about learning disorders? A recent study surveying parents of children with learning disorders in Germany revealed that only 54% of all parents receive sufficient psychoeducation and continuous support from learning therapists or educational or medical professionals following their child’s diagnostic procedure [9]. Where professionals are concerned, difficulties include choosing the suitable diagnostic measure and subsequently finding the best intervention program based on the deficit profile of the child. Parents and mental health professionals often turn to the internet in search of medical information about children’s mental health [10]. Naturally, there is a need to determine the goodness criteria for such websites and evaluate them to ensure that users get good-quality information and support.

Aiming to fill these gaps, the online platform LONDI was created by psychology and education professionals at the Clinic of the Ludwig-Maximilians-University of Munich and German Institute for International Pedagogical Research of Frankfurt [11]. LONDI offers user-specific, evidence-based information on learning disorders for different stakeholders: parents of affected children, learning therapists, school psychologists, teachers, and youth welfare workers. Apart from the information on learning disorders, it provides a unique tool for professionals (ie, learning therapists, school psychologists, and teachers) called the LONDI Help System (further referred to as HS). HS provides recommendations for evidence-based diagnostic tools and intervention programs based on a child’s specific needs.

Previous studies evaluating websites have mainly focused on the website’s content (eg, [12]) while often overlooking the website’s usage and effectiveness and whether usage is maintained over time. This study addresses this issue by adopting the RE-AIM framework for website evaluation. This framework is frequently applied in public health research for evaluating design, dissemination, and implementation of health intervention programs [12-16]. The abbreviation “RE-AIM” stands for Reach (ie, the number, proportion, and representativeness of people that the initiative reaches), Effectiveness (ie, the impact of the initiative on public health), Adoption (ie, the number and proportion of agents who are willing to use the initiative, the delivery conditions), Implementation (ie, the percentage of process objectives that were achieved and whether the intervention has been delivered as intended), and Maintenance (ie, how well the initiative is sustained over time).

A website’s *Reach* in intervention evaluations is typically assessed by the number and characteristics (eg, age) of the participants who take part in the intervention [17]. For instance, older participants rate visual aesthetics, satisfaction, perceived usefulness, and expectation confirmation more positive than younger participants, whereas there seems to be no age effect for usability [18,19]. The *Effectiveness* of a website is operationalized as any outcome intended to be changed as a result of the intervention [20]. *Implementation* is measured by how well the process objectives of the intervention are achieved and focuses on the important milestones in implementation delivery [20]. For an informational website, such a milestone is the change in the participant’s knowledge, attitudes, and self-efficacy [21,22]. *Maintenance* refers to website usage over time [20]. This can be measured by assessing users’ intention to further use a website (ie, continuance intention) and to recommend it. Building on the Technology Acceptance Model [23-25], the Information System Continuance framework [26,27], and previous evaluation studies [28-32], it is assumed that perceived usefulness and the confirmation of user expectations predict user satisfaction and continuance intention (ie, the intention to further use a website). Moreover, it is assumed that the same variables also predict the intention to recommend a website to others [33-35]. However, studies directly testing this assumption by including both outcomes (continuance intention and intention to recommend) in one model are currently missing.

In addition, previous research assessed the influence of *website characteristics* and found that content perception, usability, and visual aesthetics predict continuance intention and intention to recommend, either directly [32,35] or via satisfaction [19].

This study’s aim was to evaluate the LONDI website for two of the website’s target groups (ie, parents and learning therapists) based on 4 of the 5 RE-AIM dimensions (ie, *Reach, Effectiveness, Implementation*, and *Maintenance*). The *Adoption* dimension was not addressed, as the usage of the website by the participants was strictly guided. Although parents evaluated the informational pages developed for parents, learning therapists evaluated the informational pages for learning therapists and the HS for diagnostics and for interventions. Moreover, we aimed to create two path models to assess the factors influencing users’ intention to further use (ie, continuance intention) and the intention to recommend the website.

### Research Questions and Hypotheses

The research questions for the evaluation study were formulated according to the following RE-AIM dimensions: *Reach, Effectiveness* and *Implementation*, and *Maintenance*.

#### Reach

What is the relationship between the characteristics of the users (age, experience) and their opinions about the website characteristics (usability, content, visual aesthetics, perceived usefulness, expectations confirmation, satisfaction, continuance intention)?

Hypothesis 1a (H1a) was that opinions about the website (content, visual aesthetic, expectations confirmation, perceived usefulness, satisfaction, continuance intention) improve with age and experience for both parents and learning therapists.

Hypothesis 1b (H1b) was that opinions about the website’s usability will not depend on age or experience of the participants.

#### Effectiveness and Implementation

How does the use of the platform affect the knowledge, attitude, and self-efficacy of the target audiences?

Hypothesis 2 (H2) was that the knowledge, attitudes, and self-efficacy of parents and learning therapists will increase as a result of using the website.

#### Maintenance

How do the opinions of the target groups about the website characteristics influence their satisfaction, continuance intention, and intention to recommend the website?

To address this research question, two path models were developed and tested. In the Statistical Analysis subsection, the hypothesized and tested models (H3 and H4a and 4b) are provided, along with corresponding figures.

### Additional Data

Additionally, participants’ website ratings were compared with validated benchmarks, and open-ended qualitative data were gathered to complement our quantitative findings [36] (see the Statistical Analysis subsection for a detailed description).

### Registration

This evaluation study was preregistered on Open Science Framework [37,38].

## Methods

### Study Design

The study is a mixed methods observational study with pre- and postuse comparisons. Participants were guided by our research assistants through the website in a single 1.5-hour online session conducted via the redConnect tool. They were asked to complete specific tasks, such as read information passages or insert diagnostic data into the HS to get recommendations for intervention programs. The sessions were conducted by the research assistant staff of the university clinic who received relevant training from the study authors.

Before the session, participants completed online questionnaires about their demographic data (eg, age range, country of origin, profession; see the Measures section) and their knowledge, attitudes, and self-efficacy regarding learning disorders. During the session, parents and learning therapists familiarized themselves with the contents of the informational pages developed for parents and learning therapists, respectively. In addition to the informational pages, therapists also used both HS and were asked to find diagnostic and intervention tools based on a given case report.

After the session, participants answered the same questionnaires as before the session on their knowledge, attitudes, and self-efficacy. In addition, they completed questionnaires on the website’s usability, visual aesthetic, content perception, user satisfaction, perceived usefulness, expectations confirmation, intention to use, and intention to recommend the website.

Finally, participants also responded to an open-ended question: “What did you like about the page in particular and what would you like to improve?” This question was asked separately for the information pages, HS for diagnostics, and HS for interventions.

### Eligibility and Recruitment

Recruitment for both target groups (therapists and parents) was conducted through parent and learning therapist associations in Germany, Austria, and Switzerland. These associations typically serve as the first point of contact for parents of children with learning problems to acquire information about their child’s condition. They also function as accrediting institutions for learning therapists and provide professional training and certification. Thus, the members of these associations represent both target audiences for this study. For recruitment, we contacted the associations and asked them to send electronic study flyers to their members via their mailing lists. Flyers included information about the study aims and procedures as well as our contact email address. Participants who were interested in taking part were asked to send us an email in order to receive detailed study information and an informed consent form, which they were asked to sign and return. Once the written informed consent was received, the participants were contacted to schedule an online videocall session with one of our testers (see the Data Collection subsection for more details).

The inclusion criteria were as follows: Parents had to be a parent, a guardian, or otherwise closely participating in the upbringing of a child who either (1) had an official diagnosis of a learning disorder or (2) was experiencing severe difficulties in reading, spelling, or mathematics according to the school and the parents. Learning therapists had to be accredited in Germany, Austria, or Switzerland or be in the last semester of their respective accreditation course. The language of the website and the study was German; thus, a common inclusion criterion for both groups was to be a native or advanced German speaker.

### Ethical Considerations

The study was approved by the Ethics Committee of Ludwig-Maximilians University (LMU) of Munich on May 3, 2022 (project number 22-0300; valid until May 2, 2027). The participants signed primary informed consent forms, which included the description of the study design, its scientific basis, the study objective, conditions for participation, compensation, and information about personal data protection procedures according to the General Data Protection Regulation (GDPR) regulations. The consent form contained information about the length of the online survey that they would need to complete during the guided session. All participants received a voucher of €20 (US $23.28) upon completion of the study.

The study design was evaluated and approved by the LMU Data Protection Officer for its compliance with GDPR. The data protection measures adopted to ensure data privacy included anonymization of the data and use of GDPR-approved software for data collection (SoSci Survey, redConnect) and approved hardware for data storage (only local network computers at the LMU University Clinic).

### Measures

#### Pre-Use

The following was assessed during the pre-use evaluation: (1) demographic data, (2) knowledge about learning disorders (the number of correct answers on a true/false test), (3) attitudes toward learning disorders, and (4) self-efficacy when dealing with learning disorders.

##### Demographic Data

For parents, the following information was collected: age, gender, country of residence, diagnosis of the child (or affected learning domain), and years of experience dealing with learning disorders.

For learning therapists, the following information was collected: age, gender, country of residence, and years of professional experience.

##### Knowledge About Learning Disorders

Knowledge increase is operationalized as the increase in the number of correct answers in the knowledge test about learning disorders. The questionnaire includes 25 statement questions for each target group (parents and therapists) with the answer options “Correct,” “Incorrect,” and “Not sure.” This questionnaire was developed by authors PD and OH based on the website’s content.

##### Attitudes Toward Learning Disorders

Attitudes are defined by Ajzen and Fishbein [39] as a valent assessment of objects, concepts, or actions. According to the authors, valent assessments of subjective norms, valent assessments of behaviors, and subjective control over a certain action can all predict behavior. For our evaluation, we adapted an existing questionnaire on the attitudes of teachers toward learning disorders [40]. The questionnaire contains 24 questions for parents and 22 questions for therapists. Questions are assessed on a 6-point Likert scale. The Cronbach α reliability for the parental questionnaire based on this study’s sample was 0.71 for both the pre and posttest. For learning therapists, the Cronbach α was 0.77 for the pretest and 0.81 for the posttest. The calculation was performed using SPSS version 29 (IBM Corp).

##### Self-Efficacy Dealing With Learning Disorders

Self-efficacy change is defined as an increase in the belief that an individual can successfully execute an activity [41]. We adapted the questionnaire from Höltge et al [42]. The self-efficacy questionnaire for parents includes subscales on self-efficacy for supporting the child with academic success or school, self-efficacy for supporting the child in social life, self-efficacy for teaching the child everyday skills, and self-efficacy for emotional support. The subscales for learning therapists include their self-efficacy for reading therapy, spelling therapy, and mathematics therapy. The questionnaire for parents contains 22 questions measured on a 6-point Likert scale, while the questionnaire for learning therapists contains 36 questions. The Cronbach α for the parental questionnaire was 0.94 for the pretest and 0.93 for the posttest. The Cronbach α for the learning therapists’ questionnaire was 0.93 for the pretest and 0.93 for the posttest.

#### Postuse

The following was assessed during the postuse evaluation: (1) knowledge about learning disorders (the number of correct answers on a true/false test), (2) attitudes toward learning disorders, (3) self-efficacy for dealing with learning disorders, (4) usability, (5) visual aesthetics, (6) content perception (only for the informational pages’ evaluation), (7) perceived usefulness, (8) expectations confirmation, (9) satisfaction with the website, (10) continuance intention, and (11) intention to recommend the website.

Questionnaires 1-3 for knowledge, attitudes, and self-efficacy were the same as those applied before website use (see the descriptions in the Pre-Use subsection).

##### Usability

The international standard for computer systems ISO 9241-11 defines usability as the extent to which a product can be used by specified users to achieve specified goals with effectiveness, efficiency, and satisfaction in a specified context of use. In this study, this was measured using the German-language adaptation of the System Usability Scale [43-45], a unidimensional questionnaire that contains 10 questions assessed on an 8-point Likert scale. Reliability of the questionnaire has been reported to range between a Cronbach α of 0.85 and 0.91 [44,45].

##### Visual Aesthetics

Visual aesthetics is a measure of the aesthetic appeal as a pleasant feeling that comes before reasoning about an object [19]. A frequently used measure is the VisAWI questionnaire, developed and validated by Thielsch and Salaschek [19,45]. In this study, the full version of the questionnaire was used, with 18 questions assessed on an 8-point Likert scale: 5 questions in the Simplicity subscale, 5 questions in the Versatility subscale, 4 questions in the Colorfulness subscale, and 4 questions in the Craftsmanship subscale. The Cronbach α reliability of the questionnaire ranges between 0.76 and 0.81 [45].

##### Content Perception

Content is defined in the draft of DIN EN ISO 9241-151 as a compilation of all content objects of a web user interface [43]. We used the questionnaire developed by Theilsch and Salaschek [45] that measures 3 aspects of content perception: liking, comprehensibility, and quality/benefit. Each of these aspects is represented by 3 questions assessed on an 8-point Likert scale (9 questions in total). The reported Cronbach α reliability ranges between 0.71 and 0.90 [45].

##### Perceived Usefulness, Expectations Confirmation, Satisfaction With the Website, and Continuance Intention

The perceived usefulness of an information system is defined as the cognitive belief about how useful a system is for a user. Expectations confirmation is a measure of whether the initial expectations of using an information system have been met. Satisfaction is an affective state resulting from the experience of using a website. Continuance intention describes the intention to use an information system beyond an initial acceptance phase, in which a consumer successfully decides to start using a product [26,27]. These variables are part of the factors measured by the Information Systems Continuance Framework in the respective questionnaire [26]. The German version of the questionnaire used in this study was adapted and validated by Budner et al [46]. The questionnaire includes 13 questions assessed on an 8-point Likert-scale. The Cronbach α reliabilities for the measures are as follows: 0.92 for perceived usefulness, 0.77 for expectations confirmation, 0.92 for satisfaction, and 0.85 for continuance intention [46].

##### Intention to Recommend the Website

Intention to recommend is measured using a single-item questionnaire that is a standard for the industry: the Net Promoter Score (NPS) [47]. It measures a customer’s loyalty, which is defined as the willingness of a user to make an investment or personal sacrifice in the form of a recommendation. In our case, this is the intention of the participant (parent or therapist) to recommend the website. It is represented by one question assessed on an 11-point Likert scale (from 0 to 10).

The full-text questionnaires for all measures are available in Multimedia Appendix 1.

### Data Collection

Data were collected online using the online survey tool SoSci Survey. All questionnaires were programmed into the software. Participants were provided with a link to access the questionnaires and were required to enter their individual participant number to ensure data anonymity. All questionnaires were completed during the guided online session with the tester. The responses were saved automatically by the software on its local server in Germany and later downloaded for analysis.

### Statistical Analysis

To answer the REACH research questions (“What is the relationship between the characteristics of the users (age, experience) and their opinions about the website characteristics (usability, content, visual aesthetics, perceived usefulness, expectations confirmation, satisfaction, continuance intention)?”)*,* Pearson correlations were calculated to analyze relationships between the users’ age and experience dealing with learning disorders and their perceived usefulness of the website, website satisfaction, and continuance intention. The software used for the analysis was R version 4.4.0, and the function used was rcorr. For *P* value correction with the Bonferroni-Holm method, we used the function p.adjust (method “holm”).

Additionally, we descriptively compared participants’ ratings of all website characteristics with published benchmark scores reported in the original validation studies of the respective questionnaires. This comparison was not subjected to inference statistic testing but served as an indication whether the observed values were higher or lower than established normative values.

To answer the Effectiveness and Implementation research question (“How does the use of the platform affect the knowledge, attitude, and self-efficacy of the target audiences?”)*,* a 2-tailed paired-samples *t* test was conducted to assess the knowledge change from pre- to postuse separately for parents and learning therapists. Since attitudes and self-efficacy are reported to be linked [21,22], these 2 outcomes were analyzed in one analysis using a repeated-measures multivariate analysis of variance (MANOVA), followed by post hoc *t* tests. The analysis was done using SPSS version 29 (IBM Corp).

Finally, to answer the Maintenance research question (“How do the opinions of the target groups about the website characteristics influence their satisfaction, continuance intention, and intention to recommend the website?”), path analyses were conducted for 3 hypothesized models. These analyses were done using R version 4.4.0 with functions sem and modindices for index modification.

**Figure 1:**
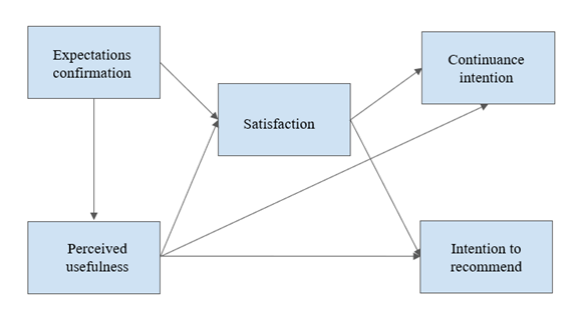
Hypothesized path model (hypothesis 3) based on the information system continuance intention framework to test the predictors of continuance intention and intention to recommend the website.

The first hypothesized model was based on the information system continuance intention framework [26,27], with the modification of adding the intention to recommend the website as a second outcome variable in addition to continuance intention. H3 tested whether expectations confirmation predicted satisfaction and perceived usefulness; perceived usefulness predicted satisfaction, continuance intention, and intention to recommend; and satisfaction predicted continuance intention and intention to recommend (see Figure 1). This model was tested 3 times: once for the informational pages using the combined data from parents and learning therapists, once for HS for diagnostics, and once for HS for interventions (HS based on the learning therapists).

**Figure 2:**
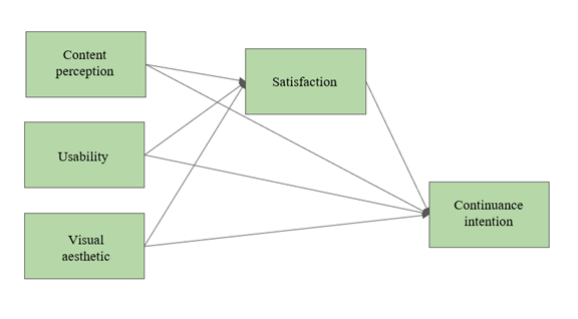
Hypothesized path model (hypothesis 4a) for the informational pages to test the predictors of satisfaction and continuance intention of parents and learning therapists.

**Figure 3:**
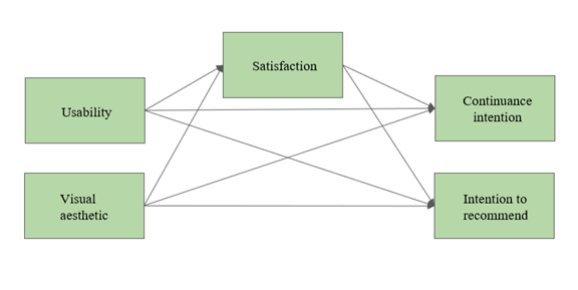
Hypothesized path model (hypothesis 4b) for the Help System (HS) tool to test the predictors of continuance intention and intention to recommend the website by learning therapists.

The second hypothesized model tested how website characteristics (content perception, usability, and visual aesthetics) predicted the continuance intention and the intention to recommend the website. Two separate models were developed: one for the informational pages and one for HS. This was done due to the varying purposes of these two parts of the website. The informational pages focus primarily on content, whereas HS is a search tool that provides links to other resources in the form of search results. Therefore, the model for the informational pages included content perception as a predictor variable, which was not part of the model for HS (see Figures 2 and 3). In order to have acceptable power for the models [48], for every path in the model, there needed to be 10 participants in the sample. Therefore, we kept the number of hypothesized paths at 7 or 8 per model due to our sample size. For the informational pages, we hypothesized (H4a) that content perception, usability, and visual aesthetics predicted continuance intention both directly and indirectly through satisfaction (Figure 2).

For the HS model, we hypothesized (H4b) that usability and visual aesthetic predicted continuance intention and intention to recommend both directly and through satisfaction (Figure 3). We tested this model 2 times: once for HS for diagnostics and once for HS for interventions.

Due to technical errors during data collection, data were missing for 3 learning therapists for the tests of attitudes and self-efficacy change (H2) and predictors of website usage (H4a and 4b), resulting in varying degrees of freedom in the reported analyses.

In addition to the quantitative analysis, a qualitative content analysis was performed on the text input received from the open-ended questions. The aim of the content analysis was to complement and explain the quantitative assessment. For the qualitative analysis, a top-down approach was adopted, derived from existing theories on website assessment [23-35] and the chosen quantitative measures [40,42,45,46]. Thus, a coding scheme was developed in advance.

The analysis consisted of the following steps. The first step involved dividing the written feedback from the participants into the smallest communicative units, each with one clear message or statement. For instance, a complete answer (“The page is well-composed...a great introductory text for beginner learning therapists...You could add some links to local websites...Fonts could be larger.”) would be divided into 4 statements: (1) “The page is well-composed...” (2) “a great introductory text for beginner learning therapists...” (3) “You could add some links to local websites...” and (4) “Fonts could be larger.”

Each statement was then coded along 3 dimensions: (1) positive, negative, or neutral; (2) existing feature or new feature request; and (3) correspondence to one of the variables used in the quantitative evaluation. This way, the previous response would be coded as follows:

1. “The page is well-composed...”—positive, existing feature, usability
2. “a great introductory text for beginner learning therapists...”—positive, existing feature, content
3. “You could add some links to local websites...”—neutral, new feature request, content
4. “Fonts could be larger”—negative, existing feature, content

Multimedia Appendix 2 contains a summary of all categories developed during coding.

## Results

### Participant Statistics

The sample consisted of 150 participants: 77 parents and 73 learning therapists (see Table 1 for detailed demographic data).

**Table 1:** Demographic data (gender, country of residence, age, and experience with learning disorders for the 2 target groups: parents and learning therapists).

Demographic data		Parents (n=77), n (%)	Learning therapists (n=73), n (%)
**Gender**			
	Female	73 (94)	68 (93)
**Country of residence**			
	Germany	73 (94)	58 (79)
	Austria	3 (3)	15 (20)
	Italy	1 (1)	0 (0)
**Age (years)**			
	25-34	0 (0)	8 (10)
	35-44	29 (39)	18 (24)
	45-54	42 (54)	29 (39)
	55-64	5 (6)	12 (16)
	≥65	1 (1)	6 (8)
**Experience with learning disorders (years)**			
	Beginner	7 (9)	10 (13)
	1-5 years	48 (62)	18 (24)
	6-10 years	18 (23)	19 (26)
	11-20 years	2 (3)	21 (28)
	>20 years	2 (3)	5 (6)

### Evaluation Outcomes

#### Benchmark Comparisons

For the informational pages, the raw scores for the website characteristics (ie, visual aesthetics, content perception, and usability) were higher than the benchmark or reference values for similar websites in German in both samples (see Table 2) [45]. For perceived usefulness, expectations confirmation, website satisfaction, and continuance intention, there are no benchmarks available; therefore, we used the results from the study by Budner et al [46] that adapted the questionnaire into German as reference values. For parents, the mean scores for perceived usefulness, expectations confirmation, website satisfaction, and continuance intention were all higher than the corresponding reference values reported by Budner et al [46]. For learning therapists, the mean scores for perceived usefulness and expectations confirmation were lower than the reference values, while satisfaction and continuance intention were higher than the reference values.

Table 3 shows the comparison between learning therapists’ opinions about HS and the same reference values taken from Budner et al [46] and Thielsch and Moshagen [45]. The usability scores for the HS for diagnostics and interventions were within the benchmark range; perceived usefulness and satisfaction scores exceeded the benchmarks, while expectations confirmation and continuance intention were slightly lower than the benchmarks.

**Table 2:** Evaluation variables for the LONDI informational pages by participant type, in comparison with available benchmarks from other studies.

Variable (raw score range)	Parents (n=77), mean (SD)	Learning therapists (n=73), mean (SD)	Benchmark or reference value, mean (SD)
Visual aesthetic (1-7)	5.14 (1.22)	5.17 (1.01)	4.08 (1.26) [45]
Content perception (1-7)	6.12 (0.97)	6.16 (0.82)	3.69 (1.17) [45]
Usability (1-100)	82.85 (17.79)	86.52 (11.73)	80-90^a^ [45]
Perceived usefulness (1-7)	5.84 (1.16)	5.03 (1.71)	5.22 (1.41) [46]
Expectations confirmation (1-7)	5.44 (1.44)	4.91 (1.51)	5.14 (1.09) [46]
Satisfaction (1-7)	5.94 (1.05)	5.94 (0.92)	5.56 (1.12) [46]
Intention to use (continuance intention)	5.87 (1.24)	5.74 (1.11)	5.50 (1.46) [46]
Intention to recommend (0-10)	9.12 (1.72)	8.86 (1.37)	N/A^b^

^a^Range 80-90 is referenced as “Good.”

^b^N/A: not available.

**Table 3:** Evaluation variables for the LONDI Help System in comparison with available benchmarks from other studies.

Variable (raw score range)	Help System for diagnostics, mean (SD)	Help System for interventions, mean (SD)	Benchmark or reference value, mean (SD)
Usability (1-100)	54.02 (13.40)	53.91 (12.61)	50-80^a^ [45]
Perceived usefulness (1-7)	5.24 (1.52)	5.29 (1.64)	5.22 (1.41) [46]
Expectations confirmation (1-7)	5.08 (1.67)	5.02 (1.73)	5.14 (1.09) [46]
Satisfaction (1-7)	5.84 (1.08)	5.60 (1.43)	5.56 (1.12) [46]
Intention to use (continuance intention)	5.34 (1.43)	5.40 (1.44)	5.50 (1.46) [46]
Intention to recommend (0-10)	8.29 (2.07)	8.10 (2.25)	N/A^b^

^a^Range 50-80 is referenced as “Acceptable.”

^b^N/A: not available.

#### Relationship Between User Demographics and Website Opinions (Reach Dimension: H1)

The relationships between user demographics and website opinions were investigated using Pearson correlation matrices. Significant *P* values were corrected for multiple comparisons. For the informational pages, no significant relationship was found between parents’ age or experience and their opinions about the website, whereas the age (but not experience) of learning therapists correlated negatively with their satisfaction with the informational pages (Table 4).

For the HS diagnostics, there was a low negative correlation between the learning therapists’ age and their opinions on the tool’s usability (see Table 5), suggesting that the older the therapists were, the more negative were their opinions on the tool’s usability. Similarly, for the HS for interventions, the opinions about the tool’s usability and satisfaction correlated negatively with learning therapists’ age.

**Table 4:** Correlations exploring the relationship between age and opinions about the website of parents (n=77) and learning therapists (n=70) for the LONDI informational pages.

Variable				Visual aesthetic		Usability		Content perception		Satisfaction		Perceived usefulness		Expectations confirmation		Continuance intention
**Parents**																
	**Age**															
		*r*	–0.06		–0.00		–0.05		–0.12		–0.21		–0.13		–0.21	
		*P* value	.57		.97		.68		.31		.07		.24		.06	
	**Experience**															
		*r*	0.11		0.12		0.06		0.00		0.09		0.08		0.01	
		*P* value	.35		.28		.59		.99		.45		.47		.95	
**Learning therapists**																
	**Age**															
		*r*	–0.29		–0.23		–0.15		–0.32^a^		–0.17		–0.12		–0.22	
		*P* value	.01		.05		.21		.006		.14		.30		.05	
		Adjusted *P* value	—^b^		—		—		.04		—		—		—	
	**Experience**															
		*r*	–0.03		–0.04		–0.12		–0.12		–0.05		–0.02		–0.04	
		*P* value	.77		.75		.30		.32		.69		.85		.76	

^a^The correlation was significant at a significance level of .05 (2-tailed) for the adjusted *P* value.

^b^Not applicable.

**Table 5:** Correlations exploring the relationship between age and website opinions of learning therapists for the Help System (HS) for diagnostics (N=70).

Variable				Usability		Perceived usefulness		Expectations confirmation		Satisfaction		Continuance intention
**HS for diagnostics**												
	**Age**											
		*r*	–0.34^a^		–0.05		–0.18		–0.22		–0.15	
		*P* value	.004		.67		.14		.06		.22	
		Adjusted *P* value	.02		—^b^		—		—		—	
	**Experience**											
		*r*	–0.15		–0.26^c^		–0.30^c^		–0.17		–0.16	
		*P* value	.20		.02		.01		.16		.17	
		Adjusted *P* value	—		.08		.05		—		—	
**HS for interventions**												
	**Age**											
		*r*	–0.37^a^		–0.13		–0.24		–0.34^a^		–0.25	
		*P* value	.001		.27		.049		.003		.04	
		Corrected *P* value	.005		—		—		.01		—	
	**Experience**											
		*r*	–0.20		–0.32		–0.30		–0.23		–0.20	
		*P* value	.09		.006		.01		.055		.09	
		Corrected *P* value	.27		.06		.08		.24		.27	

^a^The correlation was significant at a significance level of .05 (2-tailed) for the adjusted *P* value.

^b^Not applicable.

^c^The correlation was not significant at a significance level of .05 (2-tailed) for the adjusted *P* value.

#### Knowledge, Attitudes, and Self-Efficacy Change (Effectiveness and Implementation Dimensions: H2)

The comparisons between pre- and posttest performance on the knowledge test about learning disorders using paired-samples *t* tests revealed a significant increase in correct responses in both user groups (parents and learning therapists). For parents, the mean number of correct responses increased from 14.35 (SD 3.47) to 19.06 (SD 2.68; t_76_=12.02; Cohen *d*=1.37, 95% CI 1.05-1.67; *P*<.001). For learning therapists, the mean number of correct responses increased from 21.26 (SD 2.61) to 23.03 (SD 1.61; t_71_=7.03; Cohen *d*=0.83, 95% CI 0.56-1.1; *P*<.001).

The MANOVA comparing the changes in both attitudes and self-efficacy pre- and postuse revealed no significant difference between these combined variables for parents (*F*_1,76_=2.04, *P*=.14; Wilks lambda=0.95) but a significant change for learning therapists (*F*_1,68_=15.83, *P*<.001; Wilks lambda=0.68). Post hoc tests showed that the average score of learning therapists’ attitudes on a scale from 1 to 7 increased from 4.51 (SD 0.43) to 4.66 (SD 0.48; t_69_=4.81; Cohen *d*=0.27, 95% CI 0.32-0.83; *P*<.001). The average score of the learning therapists’ self-efficacy on a scale from 1 to 7 increased from 4.98 (SD 0.71) to 5.18 (SD 0.65; t_69_=4.22; Cohen *d*=0.39, 95% CI 0.25-0.75; *P*<.001).

#### Predictors of Website Usage (Maintenance Dimension: H3, H4a, H4b)

##### Continuance Intention Framework

We conducted a path analysis to test the hypothesized model (H3; Figure 1) and to examine whether the information system continuance intention framework would hold with the added outcome variable (intention to recommend). The values were centered before each analysis to correct for multicollinearity using the mutate_all function in R.

**Figure 4:**
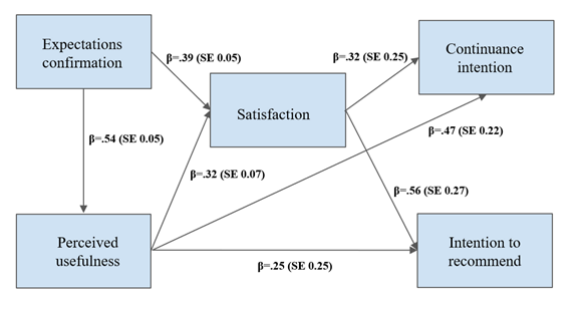
Confirmed path model (hypothesis 3) for the informational pages based on the adapted information system continuance intention framework with intention to recommend as an additional outcome variable (LONDI informational pages, N=147). All *P*<.001.

For the informational pages, the analysis based on the combined sample (parents and therapists) yielded acceptable indices of fit [17,48-52]: *χ*^2^_2_=3.1, *P*=.21; comparative fit index (CFI)=0.99, standardized root mean squared residual (SRMR)=0.02, root mean square error of approximation (RMSEA)=0.06 (see Figure 4 for the updated confirmed path model).

**Figure 5:**
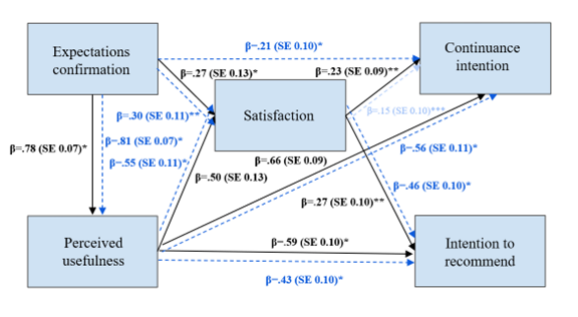
Confirmed path model for the LONDI Help System (N=70) for diagnostics (solid line) and intervention (blue dashed line) based on the adapted information system continuance intention framework with intention to recommend as an additional outcome variable. *P<.001; **P=.01; ***all other *P* values: β=.27 (SE 0.10), *P*=.007, β=.27 (SE 0.13), *P*=.04), β=.21 (SE 0.10), *P*=.03), β=.15 (SE 0.10), *P*=.13.

For the HS for diagnostics, the initial model fit yielded a significant chi-square value (*P*=.02) and acceptable indices of fit: *χ*^2^_1_=3.06, *P*=.22; CFI=0.99, SRMR=0.02, RMSEA=0.09. For the HS for interventions, the initial model fit yielded a significant chi-square value (*P*=.04). The modification indices procedure suggested adding a direct path from expectations confirmation to continuance intention. The resulting model yielded acceptable indices of fit: *χ*^2^_1_=1.6, *P*=.20; CFI=0.99, SRMR=0.01, RMSEA=0.09 (see Figure 5 for the updated confirmed path model).

##### Website Characteristics

The second hypothesized model (H4a: Figure 2; H4b: Figure 3) for the prediction of continuance intention and the intention to recommend the website proved to be oversaturated for the available data and could not be tested in the hypothesized form. Therefore, the relationships were tested in a series of regression analyses.

For the informational pages (H4a; Figure 2), the following steps were conducted: (1) multiple regression analysis of the explanatory variables (content perception, visual aesthetic, and usability) on the outcome variable (satisfaction), (2) multiple regression analysis of the explanatory variables (content perception, visual aesthetic, and usability) on the outcome variable (continuance intention), and (3) linear regression analysis of the explanatory variable (satisfaction) on the outcome variable (continuance intention).

**Figure 6:**
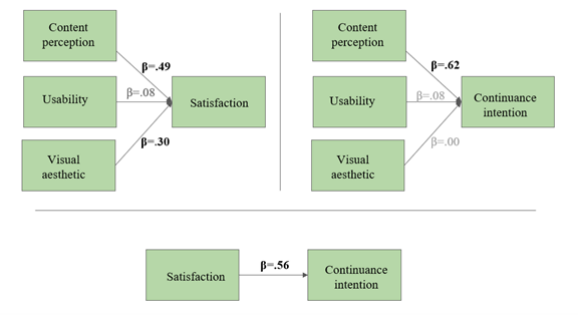
Visual representation of regression analyses of content perception, visual aesthetic, and usability on satisfaction and of satisfaction on continuance intention for LONDI informational pages (H4a; N=147).

The first step showed that visual aesthetic (*β*=.30, *P*<.001) and content perception (*β*=.49, *P*<.001) were significant predictors of website satisfaction (*R*^2^=0.59, *F*_3,143_=69.06, *P*<.001), whereas usability was not (*β*=.08, *P*=.36). The second step showed that content perception (*β*=.62, *P*<.001) was a significant predictor of continuance intention (*R*^2^=0.45, *F*_3,143_=39.74, *P*<.001), but visual aesthetics (*β*=.00, *P*=.94) and usability (*β*=.08, *P*=.42) were not. The last step revealed that satisfaction (*β*=.56, *P*<.001) was a significant predictor of continuance intention (*R*^2^=0.31, *F*_1,145_=64.29, *P*<.001). Figure 6 offers a graphic representation of the series of regressions.

For HS (H4b; Figure 3), the following steps were conducted: (1) multiple regression analysis of the explanatory variables (visual aesthetic and usability) on the outcome variable (satisfaction), (2) multivariate regression analysis of the explanatory variable (satisfaction) on the outcome variables (continuance intention and intention to recommend), and (3) multiple regression analysis of the explanatory variables (visual aesthetic and usability) on the outcome variables (continuance intention and intention to recommend).

**Figure 7:**
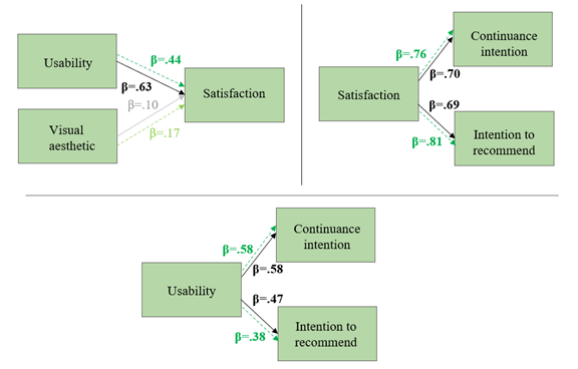
Visual representation of regression analyses of visual aesthetic and usability on satisfaction and of satisfaction on continuance intention and intention to recommend for LONDI Help System (HS) for diagnostics (solid line) and HS for interventions (green dashed line) for learning therapists (hypothesis 4b; n=70).

For HS, the first step revealed that usability (*β*=.63, *P*<.001 for diagnostics; *β*=.44, *P*<.001 for interventions) but not visual aesthetic (*β*=.10, *P*=.30 for diagnostics; *β*=.17, *P*=.14 for interventions) was a significant predictor of satisfaction (*R*^2^=0.45, *F*_2,67_=28.16, *P*<.001 for diagnostics; *R*^2^=0.27, *F*_2,67_=12.63, *P*<.001 for interventions). The second step showed that satisfaction was a significant predictor of both continuance intention (*β*=.70, *P*<.001; *R*^2^=0.49, *F*_1,68_=66.31, *P*<.001 for diagnostics; *β*=.76, *P*<.001; *R*^2^=0.58, *F*_1,68_=94.75, *P*<.001 for interventions) and intention to recommend (*β*=.69, *P*<.001; *R*^2^=0.47, *F*_1,68_=62.06, *P*<.001 for diagnostics; *β*=.81, *P*<.001; *R*^2^=0.65, *F*_1,68_=128.7, *P*<.001 for interventions). The third step showed that usability was a significant predictor of both continuance intention (*β*=.58, *P*<.001; *R*^2^=0.34, *F*_1,68_=34.57, *P*<.001 for diagnostics; *β*=.58, *P*<.001; *R*^2^=0.34, *F*_1,68_=35.11, *P*<.001 for interventions) and intention to recommend (*β*=.47, *P*<.001; *R*^2^=0.21, *F*_1,68_=19.02, *P*<.001 for diagnostics; *β*=.38, *P*<.001; *R*^2^=0.15, *F*_1,68_=12.23, *P*<.001 for interventions). Figure 7 offers a graphic representation of the series of regressions for HS diagnostics (black solid lines) and HS interventions (green dashed lines).

### Qualitative Content Analysis

In the sample of parents, a total of 414 statements about the informational pages were coded and analyzed. In the sample of learning therapists, a total of 319 statements were coded for the informational pages, 211 statements were coded for HS for diagnostics, and a further 233 statements were coded for HS for interventions.

The interrater reliability for the content analysis coding was calculated based on the first 212 units of feedback and was found to be acceptable after the first round:

1. Positive/negative: κ=0.87 (95% CI 0.81-0.93)
2. Existing feature/new feature request: κ=0.81 (95% CI 0.72-0.90)
3. Variable: κ=0.92 (95% 0.86-0.97)

Among parents, one-half of the feedback regarding the informational pages was positive (222/414, 53.6% of all statements). A further 24.4% (101/414) of the statements were neutral, and 22% (91/414) were negative. Most statements concerned existing features and referred to the content of the pages (see Table 6 for a detailed overview). Of the 378 statements about content, 201 statements (53.2%) were positive. A recurring theme in the positive feedback was the usefulness of the information (49 mentions) and the clear structure of the text (20 mentions). Negative feedback and areas of improvement mainly concerned visual aesthetics, such as legibility and insufficient contrast of fonts against the background, text length, and irritating infographic features (35 mentions). Another criticism (18 mentions) was that the procedures described on the website did not always correspond to the parents’ experiences (eg, getting support from the school or getting an official diagnosis). The most frequent new feature request was adding links with relevant information about different regions of Germany and Austria, since the school regulations regarding learning disorders vary across countries (15 mentions).

Among learning therapists, most of the feedback was again positive (194/319, 60.8% of all statements), concerned the existing features (256/319, 80.3%), and referred to the content of the pages (296/319, 92.8; see Table 6). Of the 296 statements about content, 181 (61.1%) statements were positive. The main positive issues raised included the well-structured content (41 mentions), high visual appeal (7 mentions), and ease of use of the website (8 mentions). The main criticism was that the information on the website was not perceived as useful by more experienced learning therapists, since they found it too basic (16 mentions). The new feature requests from learning therapists included adding downloadable questionnaires or survey sheets that can be used in their practice (11 mentions) and links to further professional resources (13 mentions).

The feedback given about HS by the learning therapists comprised 211 statements for the diagnostics section and 230 statements for the interventions section of the tool. Most feedback units were positive (140/211, 66.4% about diagnostics and 125/230, 54.3% about interventions). As in the case of informational pages, most feedback concerned the existing features and referred to the diagnostic and intervention measures suggested by HS (see Table 7). Approximately 20% (46/211, 21.8% for HS for diagnostics; 110/230, 47.8% for HS for interventions) of all feedback concerned usability, and another 13.3% (28/211) to 15.7% (36/230) of the feedback mentioned the perceived usefulness of the tool.

**Table 6:** Content analysis coding categories for the informational pages feedback from parents (414 statements) and learning therapists (319 statements).

Coded statements			Informational pages, n (%)		
			Parents (n=414)		Learning therapists (n=319)
**Positivity**					
	Positive statements	222 (53.6)		194 (60.8)	
	Negative statements	91 (22)		62 (19.4)	
	Neutral statements	101 (24.4)		63 (19.8)	
**Existing or new**					
	Existing	315 (76.1)		256 (80.3)	
	New	99 (23.9)		63 (19.8)	
**Variable**					
	Content	378 (91.3)		296 (92.8)	
	Visual aesthetics	3 (0.7)		1 (0.3)	
	Usability	16 (3.9)		6 (1.9)	
	Expectations confirmation	0 (0)		0 (0)	
	Satisfaction	11 (2.7)		5 (1.6)	
	Perceived usefulness	2 (0.5)		5 (1.6)	
	Continuance intention	0 (0)		0 (0)	
	Intention to recommend	1 (0.2)		1 (0.3)	
	Other	3 (0.7)		5 (1.6)	

**Table 7:** Content analysis сoding categories for the feedback on the Help System (HS) for diagnostics (211 statements) and HS for interventions (231 statements).

Coded statements		HS for diagnostics (n=211), n (%)	HS for interventions (n=230), n (%)
**Positivity**			
	Positive statements	140 (66.4)	125 (54.3)
	Negative statements	39 (18.5)	63 (27.4)
	Neutral statements	32 (15.2)	42 (18.3)
**Existing or new**			
	Existing	180 (85.3)	188 (81.7)
	New	31 (14.7)	42 (18.3)
**Variable**			
	Content	101 (47.9)	110 (47.8)
	Visual aesthetics	0 (0)	0 (0)
	Usability	46 (21.8)	46 (20)
	Expectations confirmation	0 (0)	0 (0)
	Satisfaction	21 (10)	17 (7.4)
	Perceived usefulness	28 (13.3)	36 (15.7)
	Continuance intention	6 (2.8)	5 (2.2)
	Intention to recommend	0 (0)	1 (0.4)
	Other	9 (4.3)	15 (6.5)

Among the 101 statements on the content for HS diagnostics, 58 (57.4%) were positive. Among the 46 usability statements, 33 (71%) statements were positive. Among the 110 statements on the content for HS interventions, 59 (53.6%) were positive. However, among the 46 statements on usability, only 17 (36%) were positive. The main criticisms were windows embedded within other windows, which resulted in two scroll bars on the side of the screen (8 mentions); the difficulty with getting to use the tool due to many preparatory steps (15 mentions); and overall complexity (10 mentions). The most prominent request for a new feature consisted of adding intervention programs and diagnostic measures to the databank that were not included (10 mentions).

## Discussion

### Principal Findings

The aim of this study was to evaluate the LONDI website for two of its target audiences—parents of children with learning difficulties and learning therapists—and to specify existing models for website acceptance. We therefore assessed parents’ and learning therapists’ opinions on key website characteristics, namely usability, visual aesthetics, content, perceived usefulness, expectations confirmation, continuance intention, intention to recommend, and open written feedback to complement our findings.

### Overall Acceptance of the Website

Regarding the overall acceptance of the website, both the parents’ and learning therapists’ opinions on the website were comparable to or higher than the average benchmark ratings reported for similar websites in Germany. Exceptions were the criticisms concerning perceived usefulness and expectations confirmation of the informational pages given by the learning therapists and the usability of HS. The qualitative content analysis offered further insights into these negative ratings. Learning therapists described that the information provided on the website seems more suitable for therapists with less experience. With respect to the usability of the HS, answers suggested a relatively high level of complexity.

### RE-AIM Framework

Furthermore, we assessed the Reach, Effectiveness and Implementation, and Maintenance dimensions of the RE-AIM framework and tested hypotheses regarding the influence of age and experience of the target audiences and their website opinions, knowledge, attitudes, and self-efficacy change and predictors of usage maintenance.

#### Reach

Our findings were not in line with previous research, which showed that older participants tend to rate websites more positively than younger participants, regardless of whether the website was dedicated to health topics [53]. For parents, we did not find a relationship between their age or experience and their website opinions. For learning therapists, a negative correlation was found between their age and their satisfaction with the informational pages. Furthermore, negative correlations between age and usability for both HS (diagnostics and interventions) and between age and satisfaction with the HS interventions were found. Thus, we were able to refute H1a and H1b.

One possible explanation for the inconsistent findings is the variation in complexity between the websites assessed. Research on adults’ internet usage has shown that adults 45 years and older require simpler layouts; fewer mouse movements necessary to reach a goal on the website; and a simpler design overall to account for age-related changes in vision, hearing, and psychomotor coordination [54]. Given that 63% of our learning therapists were older than 45 years, their more negative ratings may result from the relatively high complexity level of the website’s HS. A recent study by Hattink and colleagues [54] showed that simpler websites tend to be rated as more attractive and easier to use, which aligns with our findings.

In addition, the written feedback from the learning therapists provided further explanations for these negative correlations. Experienced therapists may have already established routines and preferred interventions, reducing their willingness to use new tools. Moreover, many noted that their preferred programs were not included due to the evidence-based inclusion criteria in both HS.

Together, these findings provide important information for future adaptations. Simplifying both HS and clarifying how programs are selected could improve usability and satisfaction.

#### Effectiveness and Implementation

Both groups showed increases in their knowledge about learning disorders after using the website. When it came to attitudes and self-efficacy, findings were less consistent. Although attitudes and self-efficacy increased in the sample of learning therapists, this was not the case in the sample of parents. Therefore, we can confirm H2 for learning therapists and partially confirm it for parents. The qualitative feedback provided by parents revealed several potential reasons why their attitudes and self-efficacy did not improve after using the website. Regarding attitudes, most parents in our sample already had at least 1 year of experience dealing with the learning disorders, which means that their core beliefs about the disorders have likely already been established. Furthermore, the qualitative responses highlighted the significance of parents’ self-perceptions and their perceived role in their child’s disorder. Several parents noted that realizing the disorder is not their fault and acknowledging their limited influence were particularly meaningful. Although the LONDI website does address these points, more content can be created on parents’ self-regulation, and the definition of attitudes can be broadened to include many more aspects than the disorder itself. Unfortunately, the attitudes questionnaire assessed only parents’ attitudes toward children and parent-child activities, entirely overlooking their attitudes toward themselves. Therefore, further investigation into the attitudes of parents in the context of learning disorders is needed.

Regarding self-efficacy, some parents’ feedback indicated that the website offered theory but lacked actionable content. Parents requested more practical information, such as links to local authorities and real-life examples. Some felt the website idealized how support systems function, which may explain the lack of self-efficacy gains. Thus, future adaptations should include content that better addresses the practical realities parents face.

#### Maintenance

Our analysis supported the information system continuance intention framework. Expectations confirmation and perceived usefulness remain reliable predictors of user satisfaction and continuance intention of the website. This study extended previous research by showing that the intention to recommend a website, as measured using the NPS score, is predicted by website satisfaction and perceived usefulness and might therefore be included in the information system continuance intention framework. Given that this study tested this relationship for the first time, future research would need to replicate these findings for websites with other scopes and characteristics.

Furthermore, our path analysis revealed that expectations confirmation is a direct predictor of continuance intention for HS for interventions but not for the informational pages nor HS diagnostics. In the case of HS for interventions, satisfaction is not a significant predictor of continuance intention, while expectation confirmation is. This difference may suggest that expectations confirmation plays a bigger role in user experience for online tools that have a more practical component. HS for interventions is more complex than HS for diagnostics: It requires more time to become familiar with the tool and manual input of diagnostic test results. Numerous meta-analyses done on the topic of the information system continuance intention framework have shown its effectiveness in different areas, such as mobile shopping, mobile health, online learning, and e-commerce [55-58]; however, none of these studies directly analyzed the role of involvement or level of activity of the system or tool in predicting continuance or recommendation intention. Thus, future research should investigate the degree of interactivity of an online tool and how this affects the predictors of continuance intention. Moreover, HS for interventions may be perceived by learning therapists as more important for their work, because many learning therapists do not conduct the diagnostic assessments themselves and focus solely on interventions. Therefore, expectations confirmation may play a bigger role in their continuance intention.

Regarding the website’s characteristics such as usability, content, and visual aesthetic, the results revealed that different parts of the website predicted satisfaction and continuance intention differently. For the informational pages, the website’s content and visual aesthetics were the only significant predictors of satisfaction and intention to use, while for the HS, a tool that has more interactive elements, only usability was essential. Thus, we could not confirm our hypothesis that all website characteristics will equally predict user satisfaction and continuance intention. Rather, these results suggested that the importance of the different website characteristics depends on the structure of the website itself. In line with this interpretation, previous studies have found that content and visual aesthetics were primarily important during the initial phase of forming a first impression of a website, whereas usability became more important as users interact with the website repeatedly and gain more in-depth knowledge of it [58,59]. Both HS are interactive tools for professionals that demand attention and in-depth understanding of how it works, whereas informational pages simply provide information in the form of text or graphics with few interactive elements. Therefore, future research needs to take into account that websites with different functionalities need to be assessed separately, considering their primary goal.

### Strengths and Limitations

The study’s main strength is its comprehensive scope, enabling us to assess key predictors of future user behavior and identify areas for further improvement. The website evaluation frameworks and questionnaires were chosen rigorously. We selected questionnaires that had already been validated in German to maximize the study’s validity.

However, there are also limitations to consider when interpreting our results. Participants completed the questionnaires online in the presence of a tester, potentially inducing a social desirability bias.

Another limitation concerns the questionnaire used for assessing the participants’ knowledge change. The same knowledge test was used pre- and postintervention, potentially creating a priming effect. This design was necessary due to content limitations. Regardless of this issue, the significant results suggest a positive change in knowledge after reading the platform’s content.

Finally, the reference and benchmark values that we compared our website against were only available for informational websites and not for online data banks and search tools such as the HS. Thus, it is hard to say if HS in LONDI can truly compare with other websites.

### Conclusions

Overall, the findings extended existing theories on website acceptance and provided evidence for the website’s usefulness to the target audience investigated. Moreover, they offered valuable guidance for future improvements. Although the informational pages would benefit from more practical tips for parents, the HS tool needs substantial revisions to enhance its usability. Most importantly, the results highlighted the need for a deeper understanding of the target audience and its expectations.

## Appendix

Multimedia Appendix 1 Full-text questionnaires in English and German.

Multimedia Appendix 2 Coding scheme and summary of coding categories for the qualitative content analysis.

## Abbreviations

CFI: comparative fit index
DSM: Diagnostic and Statistical Manual of Mental Disorders (Fifth Edition)
GDPR: General Data Protection Regulation
HS: Help System
LMU: Ludwig-Maximilians University
MANOVA: multivariate analysis of variance
NPS: Net Promoter Score
RMSEA: root mean square error of approximation
SRMR: standardized root mean squared residual

## Data Availability

The datasets generated or analyzed during this study are available in the Open Science Framework (OSF) repository in the project “Phase I Evaluation of the information pages of londi.de, a Website for Learning Disorders for Parents and Learning Therapists” under Files [60].
