# Supplementary material for: Evaluating a Website on Learning Disorders for Parents and Learning Therapists: Observational Mixed Methods Study
Source: JMIR Form Res. 2025 Sep 26;9:e68365. doi: 10.2196/68365 (PMC12514417; doi:10.2196/68365)
Supplement: Multimedia Appendix 1 [file formative_v9i1e68365_app1.pdf]

Multimedia Appendix 1 – Questionnaires

Knowledge – Parents

Original German version (see English translation below):

Anweisung: Bitte beantworten Sie die folgenden Fragen über Lernstörungen, indem Sie Ihre Antworten einkreisen. Wenn Sie Sich bei einer Frage unsicher sind, antworten Sie bitte mit „Unsicher“ und raten Sie nicht. Bitte lassen Sie keine Frage aus.

Die Befragung beinhaltet 25 Fragen und wird etwa **5-8 Minuten** in Anspruch nehmen.

	Richtig	Falsch	Unsicher
1. Bei einer Lernstörung handelt es sich um eine medizinische Diagnose.	☐	☐	☐
2. Anhaltenden Lernschwierigkeiten haben oft einen erschwerten Bildungsweg und psychische Belastungen zur Folge.	☐	☐	☐
3. Eine Lernstörung kann bereits im Kindergarten sicher diagnostiziert werden.	☐	☐	☐
4. Für die Feststellung einer Lernstörung ist es entscheidend, wie langanhaltend diese Schwierigkeiten bestehen.	☐	☐	☐
5. Bei Kindern mit Lernstörungen sind bestimmte Vorläuferfertigkeiten/Basiskompetenzen nicht ausreichend entwickelt.	☐	☐	☐
6. Es gibt spezifische Medikamente, die helfen, Probleme im Lesen, Schreiben oder Rechnen zu behandeln. bei Lernstörungen helfen.	☐	☐	☐
7. Aufgrund von häufigen Misserfolgen in der Schule, klagen Kinder mit Lernstörungen oft über Traurigkeit oder Bauch- und Kopfschmerzen.	☐	☐	☐

8. Eine Lese- und/oder Rechtschreibstörung tritt oft gemeinsam mit einer Rechenstörung auf.	☐ (highlighted)	☐	☐
9. Welche Formen des Nachteilsausgleichs in der Schule gewährt werden kann, hängt vom jeweiligen Bundesland ab.	☐ (highlighted)	☐	☐
10. Die Kosten für die Förderung bei einer diagnostizierten Lernstörung werden nicht von den Krankenkassen übernommen.	☐ (highlighted)	☐	☐
11. Die Diagnose einer Lernstörungen wird auf Basis des offiziellen Klassifikationsschemas für psychischer Störungen (ICD-10) gestellt.	☐ (highlighted)	☐	☐
12. Die Lese- und/oder Rechtschreibstörung sowie die Rechenstörung zählen laut der ICD-10 der WHO zu den psychischen und Verhaltensstörungen.	☐	☐ (highlighted)	☐
13. Die diagnostische Abklärung einer Lernstörung erfolgt ausschließlich durch einen Lerntherapeuten.	☐	☐ (highlighted)	☐
14. Psychische Belastungen wie Ängste oder Traurigkeit sollen bei der testpsychologischen Untersuchung von Lernstörungen untersucht werden.	☐ (highlighted)	☐	☐
15. Dokumente aus der Schule zur Lernentwicklung des Kindes spielen eine wichtige Rolle bei der Diagnosestellung.	☐ (highlighted)	☐	☐
16. Es gibt spezielle Unterrichtsmethoden für Lehrkräfte, Kinder mit einer Lernstörung im Schulunterricht zu unterstützen.	☐ (highlighted)	☐	☐
17. Ein Nachteilsausgleich aufgrund einer Lernstörung im Lesen, Rechtschreiben oder Rechnen darf nicht im Zeugnis vermerkt werden.	☐ (highlighted)	☐	☐
18. Bei einer diagnostizierten Lernstörung im Lesen, Rechtschreiben oder Rechnen reicht eine Förderung	☐	☐ (highlighted)	☐

durch die Schule oftmals aus.			
19. Informationen für die Kostenübernahme einer Lerntherapie erhalten Sie beim Jugendamt.	==☐==	☐	☐
20. Das Training des Arbeitsgedächtnisses ist ein wirksamer Bestandteil einer Lerntherapie.	☐	==☐==	☐
**Elterncoaching (Beantwortung <u>eines</u> Themenblocks pro Elternteil je nachdem, was der Proband gelesen hat), entweder:**			
**Zuhause**			
21. Sogenannte Verstärkerpläne können die Motivation von Kindern mit Lernstörungen bei den Hausaufgaben steigern.	==☐==	☐	☐
22. Spielerisches Üben im Alltag ermöglicht Eltern ihr Kind in Bezug auf seine Lernstörung zuhause zu fördern.	==☐==	☐	☐
23. Beim Einsatz von Förderprogrammen zuhause sollte darauf geachtet werden, dass nur solche angewendet werden, die das Lesen, Rechtschreiben oder Rechnen trainieren.	==☐==	☐	☐
24. Das Lesen sollte zuhause einmal pro Woche für mindestens 60 Minuten trainiert werden.	☐	==☐==	☐
25. Eltern können ihr Kind beim Hausaufgaben machen ein positives Gefühl vermitteln, indem sie es durch häufiges Fragenstellen zum Experten für das Thema machen.	==☐==	☐	☐
**oder Schule**			
21. Die Teilnahme an schulischen Zusatzangeboten (z.B. Theaterspielen, Musikunterricht) hilft sollte vermieden werden, um das Selbstwertgefühl von Kindern mit Lernstörungen zu schützen.	==☐==	☐	☐
22. Ein wichtiges Kriterium für die Auswahl einer Schule ist, dass dort Förderunterreicht für Kinder mit einer LRS angeboten wird.	==☐==	☐	☐
23. Eine Lernbegleitung kann für Kinder mit Lernstörungen eine wichtige Unterstützung im Schulalltag sein.	==☐==	☐	☐
24. Spezielle Software, die Texte in gesprochene Sprache verwandelt, kann Kinder mit Lernstörungen beim Lernen unterstützen.	==☐==	☐	☐
25. Es gibt spezielle technische Hilfsmittel, die Kinder mit Lernstörungen in der Schule unterstützen können.	==☐==	☐	☐
**oder Eltern sein**			
21. Eine Ursache von Lernstörungen sind unter	==☐==	☐	☐

anderem veränderte Prozesse im Gehirn.			
22. Die Entwicklung einer Lernstörung ist auf mangelnde Lerngelegenheiten in der Vergangenheit zurückzuführen.	☐	☐	☐
23. Es besteht ein Zusammenhang zwischen dem Stresserleben der Eltern und psychischen Belastungen der Kinder mit Lernstörungen.	☐	☐	☐
24. Es ist wichtig, Kindern mit Lernstörungen neben Fördermöglichkeiten ausreichend Zeit, z.B. für Hobbies zu geben.	☐	☐	☐
25. Telefonische Beratung zum Thema Lernstörungen wird von gesetzlichen Krankenkassen angeboten.	☐	☐	☐

English translation:

Instruction: Please answer the following questions about learning disabilities by crossing out your responses. If you are unsure about a question, please answer with "Not sure" and do not guess. Please do not leave any question unanswered.

The survey includes 25 questions and will take approximately **5-8 minutes** to complete.

	True	False	Not sure
1. A learning disorder is a medical diagnosis.	☐	☐	☐
2. Persistent learning difficulties often result in a challenging educational path and psychological stress.	☐	☐	☐
3. A learning disorder can be reliably diagnosed as early as kindergarten.	☐	☐	☐
4. For the diagnosis of a learning disorder, it is crucial to know how long the difficulties have persisted.	☐	☐	☐
5. In children with a learning disorder, certain precursor skills/basic competencies are not sufficiently developed.	☐	☐	☐
6. There are specific medications that help treat problems with reading, writing, or math in learning disorders.	☐	☐	☐
7. Due to frequent failures in school, children with learning disorders often complain of sadness, stomach	☐	☐	☐

aches or headaches.			
8. A reading and/or spelling disorder often occurs together with a math disorder.	☐	☐	☐
9. The types of compensatory measures granted in school depend on the respective federal state.	☐	☐	☐
10. The costs for the intervention in the case of a diagnosed learning disorder are not covered by health insurance.	☐	☐	☐
11. The diagnosis of a learning disorder is made based on the official classification system for mental disorders (ICD-10).	☐	☐	☐
12. According to the ICD-10 of the WHO, reading and/or spelling disorder, as well as math disorder, are classified under mental and behavioral disorders.	☐	☐	☐
13. The diagnostic evaluation of a learning disorder is carried out exclusively by a learning therapist.	☐	☐	☐
14. Psychological stress such as anxiety or sadness should be examined during the psychological testing for learning disorders.	☐	☐	☐
15. Documents from the school regarding the child's learning development play an important role in the diagnosis.	☐	☐	☐
16. There are special teaching methods for educators to support children with a learning disorder in the classroom.	☐	☐	☐
17. Compensatory measures due to a learning disorder in reading, spelling or math must not be noted on the grades report card.	☐	☐	☐
18. For a diagnosed learning disorder in reading, spelling or math, an intervention by the school is often	☐	☐	☐

sufficient.			
19. Information on the cost coverage for learning therapy can be obtained from the Youth Welfare Office.	**☐**	☐	☐
20. Training the working memory is an effective component of learning therapy.	☐	**☐**	☐
Website Section on Parents Coaching **(Each parent responds to only <u>one</u> of the three sections based on what has been read), either:**			
**At home**			
21. The so-called reinforcement plans can increase the motivation of children with learning disorders when doing homework.	**☐**	☐	☐
22. Playful practice in daily life allows parents to support their child at home with respect to their learning disorder.	**☐**	☐	☐
23. When using intervention programs at home, it should be ensured that only those programs are applied which train reading, spelling, or arithmetic.	**☐**	☐	☐
24. Reading should be practiced at home once a week for at least 60 minutes.	☐	**☐**	☐
25. Parents can give their child a positive feeling about doing homework by making them an expert on the topic through frequent questioning.	**☐**	☐	☐
**or School**			
21. Participation in extracurricular activities (e.g., theater, music lessons) should be encouraged to boost the self-esteem of children with learning disorders.	**☐**	☐	☐
22. An important criterion for choosing a school is that it offers compensatory classes for children with dyslexia.	**☐**	☐	☐
23. A special education teacher can be an important aid for children with learning disorders in their everyday school life.	**☐**	☐	☐
24. Special software that converts text into spoken language can support children with learning disorders in their learning.	**☐**	☐	☐
25. There are special technical aids that can support children with learning disorders in school.	**☐**	☐	☐
**or Being parents**			
21. One cause of learning disorders, among others, is altered processes in the brain.	**☐**	☐	☐
22. The development of a learning disorder is attributed to a lack of learning opportunities in the	☐	**☐**	☐

past.			
23. There is a correlation between the stress experienced by parents and the psychological stress of children with learning disorders.	☒	☐	☐
24. It is important to give children with learning disorders sufficient time for activities such as hobbies, in addition to intervention opportunities.	☒	☐	☐
25. Telephone counseling on the topic of learning disorders is offered by statutory health insurance companies.	☐	☒	☐

Knowledge – Learning Therapists

Original German version:

Anweisung: Bitte beantworten Sie die folgenden Fragen über Lernstörungen, indem Sie Ihre Antworten einkreisen. Wenn Sie Sich bei einer Frage unsicher sind, antworten Sie bitte mit „Unsicher“ und raten Sie nicht. Bitte lassen Sie keine Frage aus.

Die Befragung beinhaltet 25 Fragen und wird etwa **5-8 Minuten** in Anspruch nehmen.

	Richtig	Falsch	Unsicher
1. Das Jugendamt übernimmt die Kosten für Lerntherapie, wenn bei dem Kind eine drohende seelischen Behinderung vorliegen.	☒	☐	☐
2. Eine häufige psychische komorbide Störung bei Lernstörungen ist eine ADHS.	☒	☐	☐
3. In der Lerntherapie werden die gleichen Inhalte gefördert, die im Unterricht behandelt werden.	☐	☒	☐
4. Unter Notenschutz versteht man das Aussetzen der Notenvergabe in einem Bereich (z.B. Vorlesen, Rechtschreibfehler bei Diktat) in dem Schüler*innen aufgrund ihrer Lernstörung kein ausreichendes Leistungsniveau erzielen können.	☒	☐	☐
5. Matheangst entsteht häufig bei Schüler*innen als Folge ihrer nicht-erkannten Rechenstörung und den Befürchtungen, in Mathematik zu versagen.	☒	☐	☐
6. Kinder mit Lernstörungen entwickeln überzufällig häufig Ängste vor der Schule.	☒	☐	☐
7. Bei komorbid auftretenden psychischen Störungen kann es sinnvoll sein, dass zusätzlich zu einer Lerntherapie auch eine	☒	☐	☐

psychotherapeutische Behandlung durchgeführt wird.			
8. Um sich mit anderen Fachleuten, die das Kind gleichzeitig behandeln, auszutauschen, ist eine Schweigepflichtsentbindung durch die Eltern erforderlich.	☐	☐	☐
9. Oft zeigen Kinder mit Lernstörungen psychosomatische Beschwerden, wie z. B. Bauch- oder Kopfschmerzen.	☐	☐	☐
10. Das multiaxiale Klassifikationssystem für psychische Störungen des Kindes- und Jugendalters besteht aus 4 Achsen.	☐	☐	☐
11. Der Befund der die Lernstörung diagnostizierenden Stelle (meist Kinder- und Jugendpsychiatrie) ist eine wichtige Informationsquelle für die Planung der Lerntherapie.	☐	☐	☐
12. Für die Förderung bei einer Lernstörung ist eine Leistungsstanderhebung in den verschiedenen Schriftsprachkomponenten notwendig.	☐	☐	☐
13. In der Praxis ist die Anzahl der Therapiestunden, die das Jugendamt finanziert, begrenzt – auch wenn die Lese-, Rechtschreib- oder Rechenleistungen nach einer Therapie noch nicht im Durchschnittsbereich liegen.	☐	☐	☐
14. Für die Durchführung einer Lerntherapie ist die Motivation des Kindes wichtig, da ohne Motivation zur Förderung der Lernerfolg fraglich ist.	☐	☐	☐
15. Schwierige Situationen im Therapieprozess sollten im Rahmen der Supervision bearbeitet werden.	☐	☐	☐
16. Bei Konflikten zwischen Kind und Therapeut:in sollten die Eltern immer hinzugezogen werden.	☐	☐	☐
17. Die Einschätzung der Lehrkraft zum Leistungsstand des Kindes vor Beginn einer Lerntherapie kann der Therapeut:in wertvolle Anhaltspunkte zur Therapieplanung geben.	☐	☐	☐
18. Bei Verdacht auf Kindeswohlgefährdung kann die Therapeut:in eine Gefahrenmeldung an das zuständige Jugendamt machen.	☐	☐	☐
19. Die Maßnahmen zum Nachteilsausgleich umfassen methodisch-didaktische Hilfen (z. B. vergrößerte Schrift bei Vorlagen mit einer Schriftgröße >12pt)) und technische Hilfsmittel	☐	☐	☐

(z. B. Verwendung von Diktiergerät oder Computer).			
20. Laut Studien sind ca. 50% der Kinder mit einer Lernstörung von einer komorbid auftretenden psychischen Störung betroffen.	==☐==	☐	☐
21. Für die Lernverkaufsdiagnostik werden meist kurze Testverfahren eingesetzt, die wenig aufwändig für das Kind sind.	==☐==	☐	☐
22. Rechenstörung betrifft insbesondere höhere mathematische Fertigkeiten, wie Trigonometrie oder Integralrechnung, und weniger grundlegende Rechenfertigkeiten.	☐	==☐==	☐
23. Das Jugendamt ist zuständig für Informationen und Beratungen zur Eingliederungshilfe für Kinder mit Lernstörungen.	==☐==	☐	☐
24. Die Umsetzung der Lerntherapie muss bei bestehender Komorbidität mit psychischen Störungen angepasst werden.	==☐==	☐	☐
25. Ein Kriterium für die Diagnose Rechenstörung ist, dass ein unterdurchschnittliches Ergebnis im Rechentest erzielt wird.	==☐==	☐	☐

English translation:

Instruction: Please answer the following questions about learning disabilities by crossing out your responses. If you are unsure about a question, please answer with "Not sure" and do not guess. Please do not leave any question unanswered.

The survey includes 25 questions and will take approximately **5-8 minutes** to complete.

	True	False	Not sure
1. The Youth Welfare Office covers the costs of learning therapy if there is a risk of mental disability for the child.	==☐==	☐	☐
2. ADHD is a common psychological comorbid disorder in children with learning disorders.	==☐==	☐	☐
3. In learning therapy, the same learning content that is covered in class is reinforced.	☐	==☐==	☐
4. Grade protection refers to the suspension of grading in an area (e.g., reading aloud, spelling errors in dictation) where students cannot achieve an adequate level of performance due to their learning disorder.	==☐==	☐	☐

5. Math anxiety often develops in students as a result of their undiagnosed math disorder and the fear of failing in mathematics.	☐	☐	☐
6. Children with learning disorders develop school-related anxieties more often than average.	☐	☐	☐
7. For comorbid psychological disorders, it may be beneficial to provide psychotherapeutic treatment in addition to learning therapy.	☐	☐	☐
8. A confidentiality waiver from the parents is required before any communication with other professionals who are treating the child.	☐	☐	☐
9. Children with learning disorders often exhibit psychosomatic symptoms, such as stomachaches or headaches.	☐	☐	☐
10. The multiaxial classification system for mental disorders in childhood and adolescence consists of 4 axes.	☐	☐	☐
11. The report from the diagnosing institution (usually child and adolescent psychiatry) is an important source of information for planning the learning therapy.	☐	☐	☐
12. An assessment of the performance level in the various components of written language is necessary before a learning disorder intervention.	☐	☐	☐
13. In practice, the number of therapy sessions funded by the Youth Welfare Office is limited, even if reading, spelling, or arithmetic performance is still not in the average range after therapy.	☐	☐	☐
14. For the implementation of learning therapy, the child's motivation is important because, without motivation, the success of the intervention is questionable.	☐	☐	☐
15. Difficult situations in the therapy process should be addressed with the help of supervision.	☐	☐	☐
16. In case of conflicts between the child and the therapist, the parents should always be involved.	☐	☐	☐
17. The teacher's assessment of the child's performance level before the start of learning therapy can provide the therapist with valuable insights for therapy planning.	☐	☐	☐
18. If there is a suspicion of child endangerment, the therapist can report the danger to the relevant Youth Welfare Office.	☐	☐	☐

19. Measures for compensatory support include methodological-didactic aids (e.g., enlarged print on templates with a font size >12pt) and technical aids (e.g., use of a dictation device or computer).	==☐==	☐	☐
20. According to studies, approximately 50% of children with a learning disorder are affected by a comorbid psychological disorder.	==☐==	☐	☐
21. For the diagnosis of learning progress, short test procedures which are not very demanding for the child are usually used.	==☐==	☐	☐
22. A math disorder particularly affects higher mathematical skills, such as trigonometry or calculus, and less so basic arithmetic skills.	☐	==☐==	☐
23. The Youth Welfare Office is responsible for information and counseling regarding integration assistance for children with learning disorders.	==☐==	☐	☐
24. The implementation of learning therapy must be adjusted in cases of comorbid psychological disorders.	==☐==	☐	☐
25. A criterion for the diagnosis of a math disorder is that a below-average result is achieved on a math test.	==☐==	☐	☐

Attitudes towards learning disorders – Parents

Original German version:

Anweisung: Die folgenden Fragen beziehen sich auf **Ihre persönliche Haltung** gegenüber Lernstörungen. Einstellungen beruhen auf dem persönlichen Erfahrungsschatz, sind also ganz individuell und können sehr unterschiedlich ausfallen. Es gibt dabei keine richtige oder falsche Antworten.

Beantworten Sie die Fragen möglichst **spontan, offen und ehrlich**. Ihre Antworten werden **anonymisiert** erhoben, das heißt, dass Ihre Angaben sich nicht auf Ihre Person zurückführen lassen.

In welchem Maße treffen die folgenden Aussagen auf Sie zu?
Kreuzen Sie zum Antworten die Zahlen von 0 bis 5 an.

Dabei bedeutet 0, dass die Aussage für Sie gar nicht zutrifft. 5 hingegen bedeutet, dass die Aussage für Sie voll und ganz zutrifft.

		trifft gar nicht zu					trifft voll und ganz zu
1.	Lernstörungen sind ein Problem, das es bei Erwachsenen nicht gibt.	0	1	2	3	4	5
2.	Lernstörungen kommen daher, dass sich Kinder beim Lernen nicht genügend bemühen.	0	1	2	3	4	5
3.	Lernstörungen kommen daher, dass Kinder eine negative emotionale Einstellung zum Lernen haben.	0	1	2	3	4	5
4.	Kinder mit Lernstörungen könnten besser in der Schule sein, wenn sie sich mehr anstrengen.	0	1	2	3	4	5
5.	Kinder mit Lernstörungen können trotz ihrer Schwierigkeiten genauso gute Leistungen in der Schule erbringen wie Kinder ohne Lernstörungen.	0	1	2	3	4	5
6.	Kinder mit Lernstörungen haben die gleichen Bildungschancen wie Kinder ohne Lernstörungen.	0	1	2	3	4	5
7.	Kinder mit Lernstörungen sind genauso gut sozial integriert wie Kinder ohne Lernstörungen.	0	1	2	3	4	5
8.	Kinder mit Lernstörungen haben genauso viele Freund*innen wie Kinder ohne Lernstörungen.	0	1	2	3	4	5
9.	Kinder mit Lernstörungen werden von anderen Kindern ihrer Klasse weniger akzeptiert.	0	1	2	3	4	5

10.	Kinder mit Lernstörungen machen Eltern mehr Arbeit.	0 1 2 3 4 5
11.	Kinder mit Lernstörungen benötigen mehr Aufmerksamkeit von ihren Eltern.	0 1 2 3 4 5

Das Feststellen von Lernstörungen (Diagnostik) betroffener Kinder finde ich…

12.	sehr unsinnig	0	1	2	3	4	5	sehr sinnvoll
13.	sehr aussichtslos	0	1	2	3	4	5	sehr erfolgsversprechend
14.	sehr anstrengend	0	1	2	3	4	5	sehr mühelos
15.	sehr unangenehm	0	1	2	3	4	5	sehr angenehm

Die Förderung betroffener Kinder finde ich…

16.	sehr unwichtig	0	1	2	3	4	5	sehr wichtig
17.	sehr unsinnig	0	1	2	3	4	5	sehr sinnvoll
18.	sehr aussichtslos	0	1	2	3	4	5	sehr erfolgsversprechend
19.	sehr anstrengend	0	1	2	3	4	5	sehr mühelos
20.	sehr unangenehm	0	1	2	3	4	5	sehr angenehm

Wenn Sie mit Ihrem Kind zuhause lernen oder üben: Wie wahrscheinlich ist es für Sie, dass…

		sehr unwahrscheinlich					sehr wahr-scheinlich
23.	...Sie sich überfordert fühlen?	0	1	2	3	4	5
24.	…bei Ihnen Frustrationsgefühle ausgelöst werden?	0	1	2	3	4	5
25.	...Ihnen das Üben/Lernen mit Ihrem Kind mit Lernstörung Spaß macht?	0	1	2	3	4	5
26.	… das Lernen/Üben zu besseren Leistungen bei Ihrem Kindern mit Lernstörungen führen wird?	0	1	2	3	4	5

English translation:

Instruction: The following questions relate to **your personal attitude** towards learning disorders. Attitudes are based on personal experiences, so they are entirely individual and can vary greatly. There are no right or wrong answers.

Answer the questions as **spontaneously, openly, and honestly** as possible. Your responses will be collected **anonymously**, meaning that your information cannot be traced back to you personally.

To what extent do the following statements apply to you? To answer, check the numbers from 0 to 5. 0 means that the statement does not apply to you at all.

5 means that the statement fully applies to you.

		Does not apply to me					Applies fully to me
1.	Learning disorders are a problem that does not exist in adults.	0	1	2	3	4	5
2.	Learning disorders are due to children not trying hard enough to learn.	0	1	2	3	4	5
3.	Learning disorders are due to children having a negative emotional attitude towards learning.	0	1	2	3	4	5
4.	Children with learning disorders could do better in school if they tried harder.	0	1	2	3	4	5
5.	Children with learning disorders can achieve just as well in school as children without learning disorders despite their difficulties.	0	1	2	3	4	5
6.	Children with learning disorders have the same educational opportunities as children without learning disorders.	0	1	2	3	4	5
7.	Children with learning disorders are just as well socially integrated as children without learning disorders.	0	1	2	3	4	5
8.	Children with learning disorders have just as many friends as children without learning disorders.	0	1	2	3	4	5
9.	Children with learning disorders are less accepted by other children in their class.	0	1	2	3	4	5
10.	Children with learning disorders create more work for parents.	0	1	2	3	4	5
11.	Children with learning disorders need more attention from their parents.	0	1	2	3	4	5

I find diagnosing learning disorders in affected children…

12.	very useless	0	1	2	3	4	5	very useful
13.	very hopeless	0	1	2	3	4	5	very promising

14.	very difficult	0	1	2	3	4	5	very effortless
15.	very unpleasant	0	1	2	3	4	5	very pleasant

I find doing interventions with the affected children…

16.	very unimportant	0	1	2	3	4	5	very important
17.	very useless	0	1	2	3	4	5	very useful
18.	very hopeless	0	1	2	3	4	5	very promising
19.	very difficult	0	1	2	3	4	5	very effortless
20.	very unpleasant	0	1	2	3	4	5	very pleasant

When you are helping your child to study or practice at home, how probable is it that you…

		very unlikely					very likely
21.	...feel overwhelmed?	0	1	2	3	4	5
22.	…experience frustration?	0	1	2	3	4	5
23.	...will be enjoy learning with your child?	0	1	2	3	4	5
24.	… will be able to bring your child to better school performance?	0	1	2	3	4	5

Attitudes towards learning disorders – Learning Therapists

Original German version:

Instruction: Die folgenden Fragen beziehen sich auf **Ihre persönliche Haltung** gegenüber Lernstörungen. Einstellungen beruhen auf dem persönlichen Erfahrungsschatz, sind also ganz individuell und können sehr unterschiedlich ausfallen. Es gibt dabei keine richtige oder falsche Antworten.

Beantworten Sie die Fragen möglichst **spontan, offen und ehrlich**. Ihre Antworten werden **anonymisiert** erhoben, das heißt, dass Ihre Angaben sich nicht auf Ihre Person zurückführen lassen.

In welchem Maße treffen die folgenden Aussagen auf Sie zu?
Kreuzen Sie zum Antworten die Zahlen von 0 bis 5 an.

Dabei bedeutet 0, dass die Aussage für Sie gar nicht zutrifft. 5 hingegen bedeutet, dass die Aussage für Sie voll und ganz zutrifft.

		trifft gar nicht zu					trifft voll und ganz zu
1.	Kinder mit Lernstörungen könnten besser in der Schule sein, wenn sie sich mehr anstrengen würden.	0	1	2	3	4	5
2.	Kinder mit Lernstörungen können trotz ihrer Schwierigkeiten genauso gute Leistungen in der Schule erbringen wie Kinder ohne Lernstörungen.	0	1	2	3	4	5
3.	Kinder mit Lernstörungen sind genauso intelligent wie Kinder ohne Lernstörungen.	0	1	2	3	4	5
4.	Kinder mit Lernstörungen haben die gleichen Chancen auf einen guten Schulabschluss wie Kinder ohne Lernstörungen.	0	1	2	3	4	5
5.	Der reguläre Unterricht ist für Kinder mit Lernstörungen nicht geeignet, um ihre Leistungen zu verbessern.	0	1	2	3	4	5
6.	Kinder mit Lernstörungen sind genauso gut sozial integriert wie Kinder ohne Lernstörungen.	0	1	2	3	4	5
7.	Kinder mit Lernstörungen haben genauso viele Freund*innen wie Kinder ohne Lernstörungen.	0	1	2	3	4	5
8.	Kinder mit Lernstörungen werden von anderen Kindern ihrer Klasse weniger akzeptiert.	0	1	2	3	4	5

Das Feststellen von Lernstörungen (Diagnostik) betroffener Kinder finde ich…

9.	sehr uninteressant	0	1	2	3	4	5	sehr interessant
10.	sehr schwierig	0	1	2	3	4	5	sehr einfach
11.	sehr unangenehm	0	1	2	3	4	5	sehr angenehm
12.	sehr anstrengend	0	1	2	3	4	5	sehr mühelos

Die Förderung betroffener Kinder finde ich…

13.	sehr uninteressant	0	1	2	3	4	5	sehr interessant
14.	sehr schwierig	0	1	2	3	4	5	sehr einfach
15.	sehr unangenehm	0	1	2	3	4	5	sehr angenehm
16.	sehr anstrengend	0	1	2	3	4	5	sehr mühelos

Beim Feststellen von Lernstörungen (Diagnostik) und bei der Förderung betroffener Kinder: Wie wahrscheinlich ist es für Sie, dass…

		sehr unwahr-scheinlich					sehr wahr-scheinlich
17.	… es zu einer zeitaufwändigen Vorbereitung kommt?	0	1	2	3	4	5
18.	...Sie sich überfordert fühlen?	0	1	2	3	4	5
19.	…bei Ihnen Frustrationsgefühle ausgelöst werden?	0	1	2	3	4	5
20.	...Ihnen das Fördern von Kindern mit Lernstörungen Spaß macht?	0	1	2	3	4	5
21.	...Sie bei einem Konflikt mit dem Kind nicht zurechtkommen	0	1	2	3	4	5
22.	…falsche Diagnostik- oder Förderungsmethode auswählen	0	1	2	3	4	5
23.	… die Förderung zu besseren Leistungen bei den Kindern mit Lernstörungen führen wird?	0	1	2	3	4	5

English translation:

Instruction: The following questions relate to **your personal attitude** towards learning disorders. Attitudes are based on personal experiences, so they are entirely individual and can vary greatly. There are no right or wrong answers.

Answer the questions as **spontaneously, openly, and honestly** as possible. Your responses will be collected **anonymously**, meaning that your information cannot be traced back to you personally.

To what extent do the following statements apply to you? To answer, check the numbers from 0 to 5. 0 means that the statement does not apply to you at all.

5 means that the statement fully applies to you.

		Does not apply to me					Applies fully to me
1.	Children with learning disorders could do better in school if they tried harder.	0	1	2	3	4	5
2.	Children with learning disorders can achieve just as well in school as children without learning disorders despite their difficulties.	0	1	2	3	4	5

3.	Children with learning disorders are just as intelligent as children without learning disorders.	0 1 2 3 4 5
4.	Children with learning disorders have the same chances of getting a good school diploma as children without learning disorders.	0 1 2 3 4 5
5.	Regular classes are not suitable for children with learning disorders to improve their performance.	0 1 2 3 4 5
6.	Children with learning disorders are just as well socially integrated as children without learning disorders.	0 1 2 3 4 5
7.	Children with learning disorders have just as many friends as children without learning disorders.	0 1 2 3 4 5
8.	Children with learning disorders are less accepted by other children in their class.	0 1 2 3 4 5

I find diagnosing learning disorders in affected children…

9.	very uninteresting	0	1	2	3	4	5	very interesting
10.	very hard	0	1	2	3	4	5	very easy
11.	very unpleasant	0	1	2	3	4	5	very pleasant
12.	very difficult	0	1	2	3	4	5	very effortless

I find doing interventions with affected children…

13.	very uninteresting	0	1	2	3	4	5	very interesting
14.	very hard	0	1	2	3	4	5	very easy
15.	very unpleasant	0	1	2	3	4	5	very pleasant
16.	very difficult	0	1	2	3	4	5	very effortless

When diagnosing learning disorders and supporting affected children: how likely would it be for you to…

		very unlikely very likely
17.	… need a lot of time to prepare for it?	0 1 2 3 4 5
18.	... feel overwhelmed?	0 1 2 3 4 5

19.	…get frustrated?	0	1	2	3	4	5
20.	...have fun in the process?	0	1	2	3	4	5
21.	...not be able to handle a conflict with a child	0	1	2	3	4	5
22.	…you would pick the wrong methods or tests	0	1	2	3	4	5
23.	…improve the results of the child at school?	0	1	2	3	4	5

Self-efficacy – Parents

Original German version:

Instruction: Beantworten Sie die Fragen möglichst **spontan, offen und ehrlich**. Ihre Antworten werden **anonymisiert** erhoben, das heißt, dass Ihre Angaben sich nicht auf Ihre Person zurückführen lassen. Die Befragung beinhaltet 12 Fragen und wird etwa **5 Minuten** in Anspruch nehmen.

In welchem Maße treffen die folgenden Aussagen auf Sie zu?
Kreuzen Sie zum Antworten die Zahlen von 0 bis 5 an.

Dabei bedeutet 0, dass die Aussage für Sie gar nicht zutrifft. 5 hingegen bedeutet, dass die Aussage für Sie voll und ganz zutrifft.

		trifft gar nicht zu					trifft voll und ganz zu
1.	Ich traue mir zu, mein Kind mit besonderen Lernschwierigkeiten zuhause erfolgreich individuell zu fördern.						
	…bei Leseschwierigkeiten	0	1	2	3	4	5
	...bei Rechtschreibschwierigkeiten	0	1	2	3	4	5
	…bei Rechenschwierigkeiten	0	1	2	3	4	5
2.	Ich traue mir zu, mein Kind mit besonderen Lernschwierigkeiten mithilfe von veröffentlichten Trainingsprogrammen erfolgreich zu fördern.						
	…bei Leseschwierigkeiten	0	1	2	3	4	5
	...bei Rechtschreibschwierigkeiten	0	1	2	3	4	5
	…bei Rechenschwierigkeiten	0	1	2	3	4	5
3.	Ich traue mir zu, mich über wissenschaftlich basierte Möglichkeiten der Förderung von Kindern mit besonderen Lernschwierigkeiten zu informieren.						
	…bei Leseschwierigkeiten	0	1	2	3	4	5
	...bei Rechtschreibschwierigkeiten	0	1	2	3	4	5
	...bei Rechenschwierigkeiten	0	1	2	3	4	5
4.	Ich traue mir zu, meinem Kind bei den Hausaufgaben zu helfen.						
	…bei Leseschwierigkeiten	0	1	2	3	4	5

	...bei Rechtschreibschwierigkeiten	0 1 2 3 4 5
	...bei Rechenschwierigkeiten	0 1 2 3 4 5
5.	Ich traue mir zu, meinem Kind dabei zu helfen, seine schulische Leistung zu verbessern.	
	…bei Leseschwierigkeiten	0 1 2 3 4 5
	...bei Rechtschreibschwierigkeiten	0 1 2 3 4 5
	...bei Rechenschwierigkeiten	0 1 2 3 4 5
6.	Ich traue mir zu, mit meinem Kind über sein soziales Leben (Freunde, Beziehungen mit anderen) zu sprechen.	0 1 2 3 4 5
7.	Ich traue mir zu, meinem Kind bezüglich Freundschaften und Beziehungen einen guten Rat zu geben.	0 1 2 3 4 5
8.	Ich traue mir zu, mit eventuellen Situationen von Bullying oder Mobbing gut zurechtzukommen und meinem Kind dabei zu helfen.	0 1 2 3 4 5
9.	Ich traue mir zu, alltägliche Routinen meines Kindes gut zu organisieren.	0 1 2 3 4 5
10.	Ich traue mir zu, meinem Kind nützliche Fähigkeiten für das tägliche Leben beizubringen.	0 1 2 3 4 5
11.	Ich traue mir zu, das Selbstkonzept meines Kindes aufrechtzuhalten.	0 1 2 3 4 5
12.	Ich traue mir zu, eventuelle Konflikte und emotionale Probleme zuhause erfolgreich zu bewältigen.	0 1 2 3 4 5

English translation:

Instruction: The following questions relate to **your personal attitude** towards learning disorders. Attitudes are based on personal experiences, so they are entirely individual and can vary greatly. There are no right or wrong answers.

Answer the questions as **spontaneously, openly, and honestly** as possible. Your responses will be collected **anonymously**, meaning that your information cannot be traced back to you personally.

To what extent do the following statements apply to you? To answer, check the numbers from 0 to 5. 0 means that the statement does not apply to you at all.

5 means that the statement fully applies to you.

		Does not apply to me	Applies fully to me
1.	I am confident that I can successfully support my child with special learning difficulties at home.		
	...in case of reading difficulties	0 1 2 3 4 5	
	...in case of spelling difficulties	0 1 2 3 4 5	
	…in case of difficulties with arithmetics	0 1 2 3 4 5	
2.	I am confident that I can successfully support my child with special learning difficulties using published training programs.	0 1 2 3 4 5	
	...in case of reading difficulties	0 1 2 3 4 5	
	...in case of spelling difficulties	0 1 2 3 4 5	
	…in case of difficulties with arithmetics		
3.	I am confident that I can inform myself about scientifically based ways to support children with specific learning difficulties.	0 1 2 3 4 5	
	...in case of reading difficulties	0 1 2 3 4 5	
	...in case of spelling difficulties	0 1 2 3 4 5	
	…in case of difficulties with arithmetics	0 1 2 3 4 5	
4.	I am confident that I can help my child with homework.		
	...in case of reading difficulties	0 1 2 3 4 5	
	...in case of spelling difficulties	0 1 2 3 4 5	
	…in case of difficulties with arithmetics	0 1 2 3 4 5	
5.	I am confident that I can help my child improve their academic performance.		
	...in case of reading difficulties	0 1 2 3 4 5	
	...in case of spelling difficulties	0 1 2 3 4 5	
	…in case of difficulties with arithmetics	0 1 2 3 4 5	
6.	I am confident that I can talk to my child about their social life (friends, relationships with others).	0 1 2 3 4 5	
7.	I am confident that I can give my child good advice regarding friendships and relationships.	0 1 2 3 4 5	

8.	I am confident that I can effectively handle and help my child with any bullying or harassment situations.	0 1 2 3 4 5
9.	I am confident that I can organize my child's daily routines well.	0 1 2 3 4 5
10.	I am confident that I can teach my child useful skills for daily life.	0 1 2 3 4 5
11.	I am confident that I can maintain my child's self-concept.	0 1 2 3 4 5
12.	I am confident that I can successfully handle any conflicts and emotional problems at home.	0 1 2 3 4 5

Self-efficacy – Learning Therapists

Original German version:

Instruction: Beantworten Sie die Fragen möglichst **spontan, offen und ehrlich**. Ihre Antworten werden **anonymisiert** erhoben, das heißt, dass Ihre Angaben sich nicht auf Ihre Person zurückführen lassen. Die Befragung beinhaltet 12 Fragen und wird etwa **5 Minuten** in Anspruch nehmen.

In welchem Maße treffen die folgenden Aussagen auf Sie zu?
Kreuzen Sie zum Antworten die Zahlen von 0 bis 5 an.

Dabei bedeutet 0, dass die Aussage für Sie gar nicht zutrifft. 5 hingegen bedeutet, dass die Aussage für Sie voll und ganz zutrifft.

		trifft gar nicht zu … trifft voll und ganz zu
1.	Ich traue mir zu, die aktuelle Leistung des Kindes mithilfe Screenings zu überprüfen.	
	…im Lesen	0 1 2 3 4 5
	...im Rechtschreiben	0 1 2 3 4 5
	…im Rechnen	0 1 2 3 4 5
2.	Ich traue mir zu, die aktuelle Leistung des Kindes mithilfe standardisierter Testverfahren zu überprüfen.	
	…im Lesen	0 1 2 3 4 5

	...im Rechtschreiben	0 1 2 3 4 5
	…im Rechnen	0 1 2 3 4 5
3.	Ich traue mir zu, bei der Planung einer testbasierten Leistungsdiagnostik zu beurteilen, welches standardisierte Testverfahren im Individualfall hinsichtlich des Testzeitpunktes und der Testgütekriterien am besten geeignet ist.	
	…bei Leseschwierigkeiten	0 1 2 3 4 5
	...bei Rechtschreibschwierigkeiten	0 1 2 3 4 5
	…bei Rechenschwierigkeiten	0 1 2 3 4 5
4.	Ich traue mir zu, eine Lernstörung sicher zu erkennen und zu diagnostizieren, wenn sie vorliegt.	
	…bei Leseschwierigkeiten	0 1 2 3 4 5
	...bei Rechtschreibschwierigkeiten	0 1 2 3 4 5
	…bei Rechenschwierigkeiten	0 1 2 3 4 5
5.	Ich traue mir zu, mich über die wissenschaftlich basierten Diagnoseverfahren informieren zu können.	
	…bei Leseschwierigkeiten	0 1 2 3 4 5
	...bei Rechtschreibschwierigkeiten	0 1 2 3 4 5
	…bei Rechenschwierigkeiten	0 1 2 3 4 5
6.	Ich traue mir zu, mich über wissenschaftlich basierte Möglichkeiten der Förderung von Kindern mit Lernstörungen zu informieren.	
	…bei Leseschwierigkeiten	0 1 2 3 4 5
	...bei Rechtschreibschwierigkeiten	0 1 2 3 4 5
	…bei Rechenschwierigkeiten	0 1 2 3 4 5
7.	Ich traue mir zu, geeignete Förderverfahren für individuelle Förderung auszuwählen.	
	…bei Leseschwierigkeiten	0 1 2 3 4 5
	...bei Rechtschreibschwierigkeiten	0 1 2 3 4 5
	…bei Rechenschwierigkeiten	0 1 2 3 4 5
8.	Ich traue mir zu, Kinder mit Lernstörungen erfolgreich individuell und mithilfe von evidenzbasierten Trainingsprogrammen zu fördern.	
	…bei Leseschwierigkeiten	0 1 2 3 4 5

	...bei Rechtschreibschwierigkeiten	0 1 2 3 4 5
	…bei Rechenschwierigkeiten	0 1 2 3 4 5
9.	Ich traue mir zu, das Selbstkonzept von Kindern mit Lernstörungen effektiv aufzubauen.	
	…bei Leseschwierigkeiten	0 1 2 3 4 5
	...bei Rechtschreibschwierigkeiten	0 1 2 3 4 5
	…bei Rechenschwierigkeiten	0 1 2 3 4 5
10.	Ich traue mir zu, die Lernmotivation von Kindern mit Lernstörungen bedeutsam zu steigern.	
	…bei Leseschwierigkeiten	0 1 2 3 4 5
	...bei Rechtschreibschwierigkeiten	0 1 2 3 4 5
	…bei Rechenschwierigkeiten	0 1 2 3 4 5
11.	Ich traue mir zu, die Eltern von Kindern mit einer Lernstörung in Bezug auf Ursachen, Symptome, Verlauf entsprechend dem wissenschaftlichen Erkenntnisstand zu beraten.	
	…bei Leseschwierigkeiten	0 1 2 3 4 5
	...bei Rechtschreibschwierigkeiten	0 1 2 3 4 5
	…bei Rechenschwierigkeiten	0 1 2 3 4 5
12.	Ich traue mir zu, Eltern von Kindern mit Lernstörungen über die gesetzlichen Regelungen und die Möglichkeiten einer Kostenübernahme für eine außerschulische Lerntherapie aufzuklären.	
	…bei Leseschwierigkeiten	0 1 2 3 4 5
	...bei Rechtschreibschwierigkeiten	0 1 2 3 4 5
	…bei Rechenschwierigkeiten	0 1 2 3 4 5

English translation:

Instruction: The following questions relate to **your personal attitude** towards learning disorders. Attitudes are based on personal experiences, so they are entirely individual and can vary greatly. There are no right or wrong answers.

Answer the questions as **spontaneously, openly, and honestly** as possible. Your responses will be collected **anonymously**, meaning that your information cannot be traced back to you personally.

To what extent do the following statements apply to you? To answer, check the numbers from 0 to 5. 0 means that the statement does not apply to you at all.

5 means that the statement fully applies to you.

		trifft gar nicht zu / trifft voll und ganz zu
1.	I am confident that I can check the child's current performance using screenings.	
	…in reading	0 1 2 3 4 5
	...in spelling	0 1 2 3 4 5
	…in arithmetics	0 1 2 3 4 5
2.	I am confident that I can check the child's current performance using standardized tests.	
	…in reading	0 1 2 3 4 5
	...in spelling	0 1 2 3 4 5
	…in arithmetics	0 1 2 3 4 5
3.	I am confident that I can assess which standardized test procedure is most suitable in an individual case for planning a test-based performance diagnosis, considering the timing and quality criteria of the test.	
	...in case of reading difficulties	0 1 2 3 4 5
	...in case of spelling difficulties	0 1 2 3 4 5
	…in case of difficulties with arithmetics	0 1 2 3 4 5
4.	I am confident that I can reliably recognize and diagnose a learning disorder if it is present.	
	...in case of reading difficulties	0 1 2 3 4 5
	...in case of spelling difficulties	0 1 2 3 4 5
	…in case of difficulties with arithmetics	0 1 2 3 4 5
5.	I am confident that I can inform myself about scientifically based diagnostic procedures.	
	...in case of reading difficulties	0 1 2 3 4 5

	...in case of spelling difficulties	0 1 2 3 4 5
	…in case of difficulties with arithmetics	0 1 2 3 4 5
6.	I am confident that I can inform myself about scientifically based methods for supporting children with learning disorders.	
	...in case of reading difficulties	0 1 2 3 4 5
	...in case of spelling difficulties	0 1 2 3 4 5
	…in case of difficulties with arithmetics	0 1 2 3 4 5
7.	I am confident that I can select appropriate intervention methods for individual support.	
	...in case of reading difficulties	0 1 2 3 4 5
	...in case of spelling difficulties	0 1 2 3 4 5
	…in case of difficulties with arithmetics	0 1 2 3 4 5
8.	I am confident that I can successfully support children with learning disorders individually and with evidence-based training programs.	
	...in case of reading difficulties	0 1 2 3 4 5
	...in case of spelling difficulties	0 1 2 3 4 5
	…in case of difficulties with arithmetics	0 1 2 3 4 5
9.	I am confident that I can effectively build the self-concept of children with learning disorders.	
	...in case of reading difficulties	0 1 2 3 4 5
	...in case of spelling difficulties	0 1 2 3 4 5
	…in case of difficulties with arithmetics	0 1 2 3 4 5
10.	I am confident that I can significantly increase the learning motivation of children with learning disorders.	
	...in case of reading difficulties	0 1 2 3 4 5
	...in case of spelling difficulties	0 1 2 3 4 5
	…in case of difficulties with arithmetics	0 1 2 3 4 5
11.	I am confident that I can advise the parents of children with learning disorders on the causes, symptoms, and progression according to the current scientific knowledge.	
	...in case of reading difficulties	0 1 2 3 4 5

	...in case of spelling difficulties	0 1 2 3 4 5
	…in case of difficulties with arithmetics	0 1 2 3 4 5
12.	I am confident that I can inform parents of children with learning disorders about the legal regulations and the possibilities of cost coverage for extracurricular learning therapy.	
	...in case of reading difficulties	0 1 2 3 4 5
	...in case of spelling difficulties	0 1 2 3 4 5
	…in case of difficulties with arithmetics	0 1 2 3 4 5

Usability – Parents/Learning Therapists

Original German version:

Instruction: Jetzt bekommen Sie die Aussagen, die Ihre **persönliche Meinung zu verschiedenen Aspekten** der Website Londi und des Hilfssystems widerspiegeln. Beantworten Sie die Fragen möglichst spontan, offen und ehrlich. Ihre Antworten werden anonymisiert erhoben, das heißt, dass Ihre Angaben sich nicht auf Ihre Person zurückführen lassen. Die Befragung wird etwa 6 Minuten in Anspruch nehmen.

In welchem Maße treffen die folgenden Aussagen auf Sie zu?
Kreuzen Sie zum Antworten die Zahlen von **0 bis 7** an.

		trifft gar nicht zu							trifft voll und ganz zu
1.	Ich denke, dass ich die Londi-Webseite gerne häufig benutzen würde.	0	1	2	3	4	5	6	7
2.	Ich fand die Webseite unnötig komplex.	0	1	2	3	4	5	6	7
3.	Ich fand die Londi-Webseite einfach zu benutzen.	0	1	2	3	4	5	6	7
4.	Ich glaube, ich würde die Hilfe einer technisch versierten Person benötigen, um die Londi-Webseite benutzen zu können.	0	1	2	3	4	5	6	7
5.	Ich fand, die verschiedenen Funktionen der LONDI-Webseite waren gut integriert.	0	1	2	3	4	5	6	7
6.	Ich denke, die LONDI-Webseite enthielt zu viele Inkonsistenzen.	0	1	2	3	4	5	6	7
7.	Ich kann mir vorstellen, dass die meisten Menschen den Umgang mit der LONDI-Webseite sehr schnell lernen.	0	1	2	3	4	5	6	7
8.	Ich fand die LONDI-Webseite sehr umständlich zu nutzen.	0	1	2	3	4	5	6	7
9.	Ich fühlte mich bei der Benutzung der LONDI-Webseite sehr sicher.	0	1	2	3	4	5	6	7
10.	Ich musste eine Menge lernen, bevor ich anfangen konnte die LONDI-Webseite zu verwenden.	0	1	2	3	4	5	6	7

English translation:

Instruction to all the following questionnaires: Now you will see statements reflecting your **personal opinion on various aspects** of the Londi website and its help system. Please answer the questions as spontaneously, openly, and honestly as possible. Your responses will

be collected anonymously, meaning that your information cannot be traced back to you personally. The survey will take approximately 6 minutes to complete.

To what extent do the following statements apply to you? To answer, check the numbers from **0 to 7.**

		Does not apply to me							Applies fully to me
1.	I think that I would like to use the Londi website frequently.	0	1	2	3	4	5	6	7
2.	I found the website unnecessarily complex.	0	1	2	3	4	5	6	7
3.	I found the Londi website easy to use.	0	1	2	3	4	5	6	7
4.	I believe I would need the help of a technically savvy person to use the Londi website.	0	1	2	3	4	5	6	7
5.	I found the different functions of the Londi website well integrated.	0	1	2	3	4	5	6	7
6.	I think the Londi website had too many inconsistencies.	0	1	2	3	4	5	6	7
7.	I can imagine that most people would learn to use the Londi website very quickly.	0	1	2	3	4	5	6	7
8.	I found the Londi website very cumbersome to use.	0	1	2	3	4	5	6	7
9.	I felt very confident using the Londi website	0	1	2	3	4	5	6	7
10.	I had to learn a lot before I could start using the Londi website.	0	1	2	3	4	5	6	7

Visual Aesthetics – Parents/Learning Therapists

Original German version:

		trifft gar nicht zu							trifft voll und ganz zu
1.	Das Layout* wirkt zu gedrängt.<br>*Unter Layout versteht man den allgemeinen strukturellen Aufbau der Webseite und deren Aussehen.	0	1	2	3	4	5	6	7
2.	Das Layout ist gut zu erfassen.	0	1	2	3	4	5	6	7
3.	Das Layout erscheint angenehm gegliedert.	0	1	2	3	4	5	6	7
4.	Die Seite erscheint zu uneinheitlich.	0	1	2	3	4	5	6	7
5.	Auf der Seite passt alles zusammen.	0	1	2	3	4	5	6	7
6.	Die Seitengestaltung ist uninteressant.	0	1	2	3	4	5	6	7
7.	Das Layout ist originell.	0	1	2	3	4	5	6	7
8.	Die Gestaltung wirkt einfallslos.	0	1	2	3	4	5	6	7
9.	Das Layout wirkt dynamisch.	0	1	2	3	4	5	6	7
10.	Das Layout ist angenehm vielseitig.	0	1	2	3	4	5	6	7
11.	Die farbliche Gesamtgestaltung wirkt attraktiv.	0	1	2	3	4	5	6	7
12.	Die Farben passen nicht zueinander.	0	1	2	3	4	5	6	7
13.	Der Farbeinsatz ist nicht gelungen.	0	1	2	3	4	5	6	7
14.	Die Farben haben eine angenehme Wirkung.	0	1	2	3	4	5	6	7
15.	Das Layout ist professionell.	0	1	2	3	4	5	6	7
16.	Das Layout ist nicht zeitgemäß.	0	1	2	3	4	5	6	7
17.	Die Seite erscheint mit Sorgfalt gemacht	0	1	2	3	4	5	6	7
18.	Das Layout wirkt konzeptlos.	0	1	2	3	4	5	6	7

English translation:

		Does not apply to me							Applies fully to me
1.	The layout* appears too cramped. *Layout refers to the overall structural design and appearance of the website.	0	1	2	3	4	5	6	7
2.	The layout is easy to grasp.	0	1	2	3	4	5	6	7
3.	The layout appears pleasantly organized.	0	1	2	3	4	5	6	7
4.	The page seems too inconsistent.	0	1	2	3	4	5	6	7
5.	Everything on the page fits together.	0	1	2	3	4	5	6	7

6.	The page design is uninteresting.	0	1	2	3	4	5	6	7
7.	The layout is original.	0	1	2	3	4	5	6	7
8.	The design appears uninspired.	0	1	2	3	4	5	6	7
9.	The layout looks dynamic.	0	1	2	3	4	5	6	7
10.	The layout is pleasantly varied.	0	1	2	3	4	5	6	7
11.	The overall color scheme looks attractive.	0	1	2	3	4	5	6	7
12.	The colors do not match each other.	0	1	2	3	4	5	6	7
13.	The use of color is not successful.	0	1	2	3	4	5	6	7
14.	The colors have a pleasant effect.	0	1	2	3	4	5	6	7
15.	The layout is professional.	0	1	2	3	4	5	6	7
16.	The layout is not contemporary.	0	1	2	3	4	5	6	7
17.	The page appears to be made with care.	0	1	2	3	4	5	6	7
18.	The layout seems to have no concept.	0	1	2	3	4	5	6	7

Content Perception – Parents/Learning Therapists

Original German version:

		trifft gar nicht zu							trifft voll und ganz zu
1.	Die Webseite weckt mein Interesse.	0	1	2	3	4	5	6	7
2.	Der Inhalt der Webseite gefällt mir.	0	1	2	3	4	5	6	7
3.	Ich lese diese Webseite gerne.	0	1	2	3	4	5	6	7
4.	Die einzelnen Sätze sind einfach zu lesen.	0	1	2	3	4	5	6	7
5.	Die Texte liefern mir kurz und bündig die wichtigsten Informationen.	0	1	2	3	4	5	6	7
6.	Der Sprachgebrauch in den Texten ist geläufig und allgemein verständlich.	0	1	2	3	4	5	6	7
7.	Die Informationen sind qualitativ hochwertig.	0	1	2	3	4	5	6	7
8.	Ich finde die Informationen auf der Webseite nützlich.	0	1	2	3	4	5	6	7
9.	Die Inhalte der Webseite erscheinen mir so wichtig, dass ich sie mir ausdrucken oder speichern würde.	0	1	2	3	4	5	6	7

English translation:

		Does not apply to me							Applies fully to me
1.	The website arouses my interest.	0	1	2	3	4	5	6	7
2.	I like the content of the website.	0	1	2	3	4	5	6	7
3.	I enjoy reading this website.	0	1	2	3	4	5	6	7
4.	The individual sentences are easy to read.	0	1	2	3	4	5	6	7
5.	The texts provide me with the most important information concisely.	0	1	2	3	4	5	6	7
6.	The language used in the texts is familiar and generally understandable.	0	1	2	3	4	5	6	7
7.	The information is of high quality.	0	1	2	3	4	5	6	7
8.	I find the information on the website useful.	0	1	2	3	4	5	6	7
9.	The content of the website seems important enough for me to print or save.	0	1	2	3	4	5	6	7

Information System Continuance Intention – Parents

Original German version:

1.	Ich habe vor, Londi weiterhin zu nutzen.	0 1 2 3 4 5 6 7
2.	Ich würde Londi anderen ähnlichen Seiten zu Lernstörungen vorziehen.	0 1 2 3 4 5 6 7
3.	In der Zukunft werde ich Londi wahrscheinlich nicht mehr nutzen.	0 1 2 3 4 5 6 7
4.	Die Nutzung von Londi wird mir beim alltäglichen Umgang mit dem Thema Lernstörungen helfen.	0 1 2 3 4 5 6 7
5.	Die Nutzung von Londi wird mein Wissen über Lernstörungen verbessern.	0 1 2 3 4 5 6 7
6.	Ich finde die praktischen Übungen und Strategien im Teil „Elterncoaching" nützlich, um mein Kind zuhause individuell zu fördern.	0 1 2 3 4 5 6 7
7.	Insgesamt sehe ich Londi als eine nützliche Seite für meinen Umgang mit Lernstörungen.	0 1 2 3 4 5 6 7
8.	Meine Erfahrungen mit der Nutzung von Londi waren besser als erwartet.	0 1 2 3 4 5 6 7
9.	Die Informationen auf Londi fand ich besser als ich erwartet hatte.	0 1 2 3 4 5 6 7
10.	Insgesamt wurden die meisten meiner Erwartungen an Londi erfüllt.	0 1 2 3 4 5 6 7

Wie denken Sie über **Ihre Erfahrungen mit der Nutzung** von Londi:

11.	sehr frustriert	0	1	2	3	4	5	6	7	hochzufrieden
12.	sehr verärgert	0	1	2	3	4	5	6	7	sehr erfreut
13.	überhaupt nicht überzeugt	0	1	2	3	4	5	6	7	sehr überzeugt

English translation:

1.	I intend to continue using Londi.	0 1 2 3 4 5 6 7
2.	I prefer Londi to other similar pages about learning disorders.	0 1 2 3 4 5 6 7
3.	I will probably not use Londi in the future.	0 1 2 3 4 5 6 7

4.	Londi will help me in my daily life when it comes to dealing with learning disorders.	0 1 2 3 4 5 6 7
5.	Using Londi will help me improve my knowledge about learning disorders.	0 1 2 3 4 5 6 7
6.	I find the practical exercises and strategies in „Parents Coaching“ useful when it comes to supporting my child at home.	0 1 2 3 4 5 6 7
7.	Altogether I see Londi as a useful page for dealing with learning disorders.	0 1 2 3 4 5 6 7
8.	My experience using Londi was better than expected.	0 1 2 3 4 5 6 7
9.	The information provided on the website was better than expected.	0 1 2 3 4 5 6 7
10.	Altogether most of my expectations of Londi have been fulfilled.	0 1 2 3 4 5 6 7

What do you think about **your experience using Londi**:

11.	very frustrated	0	1	2	3	4	5	6	7	very pleased
12.	very angry	0	1	2	3	4	5	6	7	very happy
13.	absolutely not convinced	0	1	2	3	4	5	6	7	very convinced

Information System Continuance Intention – Learning Therapists

Original German version:

1.	Ich habe vor, Londi weiterhin zu nutzen.	0 1 2 3 4 5 6 7
2.	Ich würde Londi anderen ähnlichen Seiten zu Lernstörungen vorziehen.	0 1 2 3 4 5 6 7
3	Es ist wahrscheinlich, dass ich Londi langfristig nutzen werde.	0 1 2 3 4 5 6 7
4.	Die Nutzung von Londi verbessert meine Leistungsfähigkeit bei der Arbeit mit Lernstörungen.	0 1 2 3 4 5 6 7
5.	Die Nutzung von Londi erhöht meine Produktivität.	0 1 2 3 4 5 6 7
6.	Die Nutzung von Londi fördert meine Effektivität bei der Arbeit mit betroffenen Kindern.	0 1 2 3 4 5 6 7
7.	Insgesamt sehe ich Londi als nützliches Tool für meine Arbeit als Lerntherapeut*in.	0 1 2 3 4 5 6 7

8.	Meine Erfahrungen mit der Nutzung von Londi waren besser als erwartet.	0 1 2 3 4 5 6 7
9.	Der Wert von Londi war für mich höher als erwartet.	0 1 2 3 4 5 6 7
10.	Insgesamt wurden die meisten meiner Erwartungen an Londi erfüllt.	0 1 2 3 4 5 6 7

Wie denken Sie über **Ihre Erfahrungen mit der Nutzung** von Londi:

11.	sehr frustriert	0	1	2	3	4	5	6	7	hochzufrieden
12.	sehr verärgert	0	1	2	3	4	5	6	7	sehr erfreut
13.	überhaupt nicht überzeugt	0	1	2	3	4	5	6	7	sehr überzeugt

English translation:

1.	I intend to continue using Londi.	0 1 2 3 4 5 6 7
2.	I prefer Londi to other similar pages about learning disorders.	0 1 2 3 4 5 6 7
3.	I will probably not use Londi in the future.	0 1 2 3 4 5 6 7
4.	Londi increases my performance in my work with learning disorders.	0 1 2 3 4 5 6 7
5.	Using Londi improves my productivity.	0 1 2 3 4 5 6 7
6.	Using Londi makes me more efficient in my work with affected children.	0 1 2 3 4 5 6 7
7.	Altogether I see Londi as a useful page for my work as a learning therapist.	0 1 2 3 4 5 6 7
8.	My experience using Londi was better than expected.	0 1 2 3 4 5 6 7
9.	The value of Londi for me is higher than expected.	0 1 2 3 4 5 6 7
10.	Altogether most of my expectations of Londi have been fulfilled.	0 1 2 3 4 5 6 7

What do you think about **your experience using Londi**:

11.	very frustrated	0	1	2	3	4	5	6	7	very pleased
12.	very angry	0	1	2	3	4	5	6	7	very happy
13.	absolutely not convinced	0	1	2	3	4	5	6	7	very convinced
