# Supplementary material for: Evaluating a Website on Learning Disorders for Parents and Learning Therapists: Observational Mixed Methods Study
Source: JMIR Form Res. 2025 Sep 26;9:e68365. doi: 10.2196/68365 (PMC12514417; doi:10.2196/68365)
Supplement: Multimedia Appendix 2 [file formative_v9i1e68365_app2.pdf]

Coding scheme used to categorize feedback of parents and learning therapists

Each statement must be coded according to 3 dimensions: Positivity, Newness, and Website Aspect:

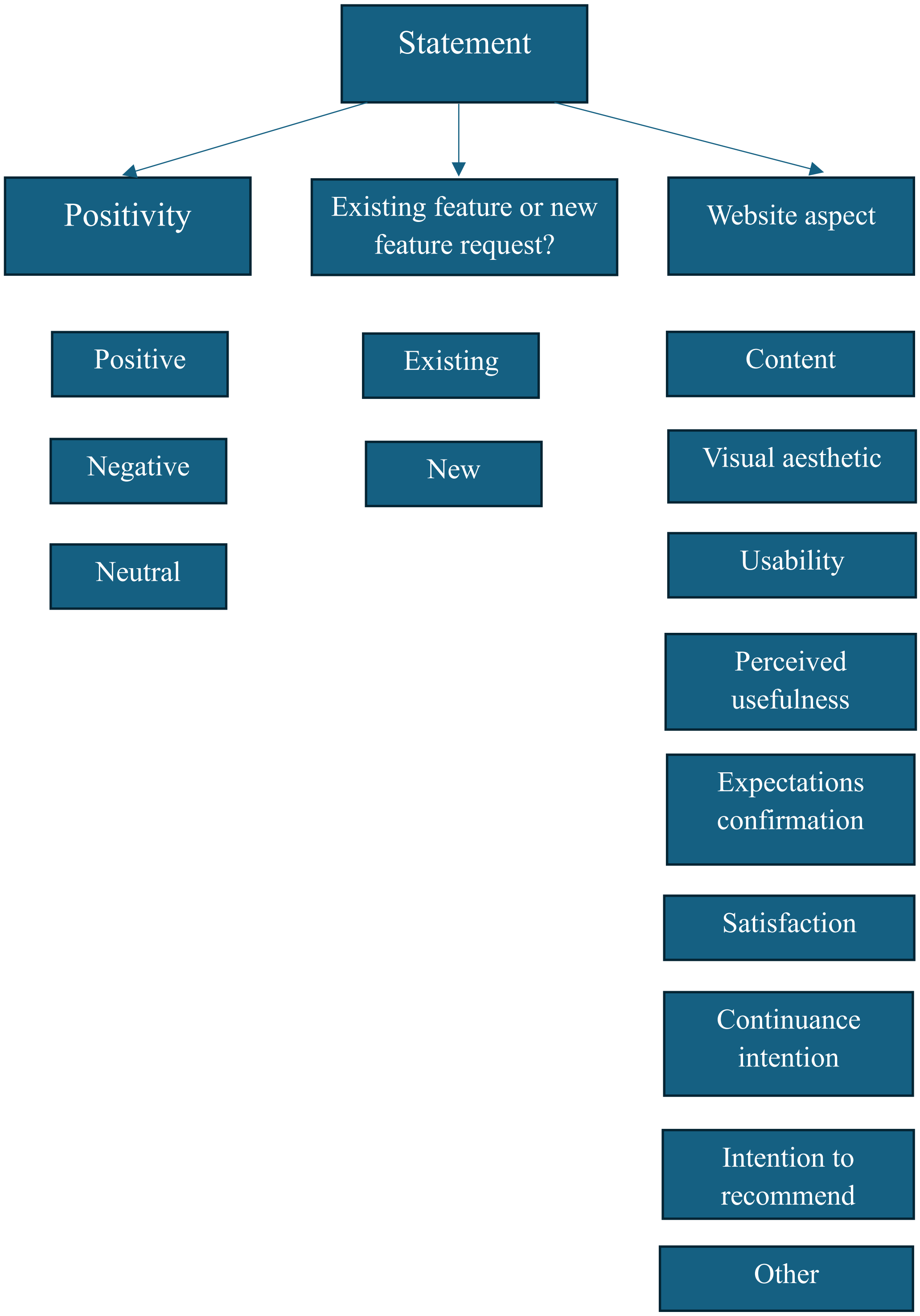

Each piece of feedback must be coded **three times** (if the feedback concerns content, then **four times**):

1. **Is the feedback positive, negative, or neutral?**
   If someone wishes for something that doesn't yet exist on the site, or if information is missing, it is considered **neutral**.
   Only feedback on elements that are **already present** on the site can be considered **positive** or **negative**.
2. **Is the feedback about existing features/content, or is it a request for new features that are not yet on the platform?**
   If someone says *"I'm missing…"* or *"I would like to have…"*, it is marked as **new**.
3. **Which variable does the feedback relate to?**

Content refers generally to everything related to **text and information**. This includes anything related to the **accuracy, correctness, or completeness of the information**, for example, whether information is missing. It also includes comments on **infographics, glossaries, font size or color**.

Usability is defined as the **general quality of how well a system suits its intended purpose**. This includes how **user-friendly** the site is, how easy it is to use and navigate, and whether everything is easy to find and accessible. Examples include:

- Buttons,
- Scrolling,
- Menu items, etc.

Exception: Comments on **text readability, fonts**, or anything text-related belong to **Content**, even if they affect usability, because they concern the **presentation of information**, not the system design.

Visual aesthetics refers to the **aesthetic appeal** as a **pleasant emotional response before any rational evaluation** of an object. This includes **subjective, emotional impressions** of the website's design — whether it is considered beautiful or appealing.

⚠️ **Important**: Distinguish whether the comment is about **the overall site** (→ **Visual Aesthetics**) or about **specific elements** like graphics or texts (→ **Content – Infographics** or **Content – Information**).

Perceived usefulness of an information system refers to the **user's belief** about how useful the system is.

Statements like *"I found the website very useful"* count as **Perceived Usefulness**.
But if someone says *"I found this information particularly useful"*, it counts as **Content – Information**.

Satisfaction is an **emotional state** resulting from the **experience** of using the website. You may code a statement as **Satisfaction** if it refers to the **overall experience** with the site.
Example: *"I thought the information was great"* → **Content – Information**, not Satisfaction.

Confirmation of expectations refers to whether the user's **initial expectations** about using the site were met.
All statements relating to **expectations** fall under this category.

Continuance intention describes the **intention to continue using** the website beyond the initial experience.
This includes statements like:

- *"I will continue to use it"*,
- *"I will recommend it to others"*,
- *"Please advertise this site everywhere"* → This last one would be **Intention to Recommend**.

Other refers to **political statements**, **general wishes**, or **comments about real-life situations in Germany** that are **not related to the website itself**. These are coded as **Neutral – New – Other**.
